# Innovative approaches in precision radiation oncology: advanced imaging technologies and challenges which shape the future of radiation therapy

**DOI:** 10.3389/fmed.2025.1686593

**Published:** 2025-10-30

**Authors:** Yue Yan, Daniel A. Alexander, Bryan P. Bednarz, Lawrence F. Bronk, Huixiao Chen, David J. Gladstone, Bin Han, Christopher M. Iannuzzi, Yuting Li, Ngoc Nguyen, Natasha Mulenga, Natalie N. Viscariello, Yuenan Wang, Joseph Weygand, Yana Zlateva, Fada Guan

**Affiliations:** 1 Department of Radiation Oncology & Applied Sciences, Dartmouth-Hitchcock Medical Center, Lebanon, NH, United States; 2 Geisel School of Medicine, Dartmouth College, Hanover, NH, United States; 3 Thayer School of Engineering, Dartmouth College, Hanover, NH, United States; 4 Department of Medical Physics, University of Wisconsin Madison, Madison, WI, United States; 5 Department of Radiation Physics, University of Texas MD Anderson Cancer Center, Houston, TX, United States; 6 Department of Therapeutic Radiology, Yale University School of Medicine, New Haven, CT, United States; 7 Department of Radiation Oncology, Stanford University, Stanford, CA, United States; 8 Hartford Health Care Cancer Institute, St. Vincent's Medical Center, Bridgeport, CT, United States; 9 Department of Radiation Oncology, Heersink School of Medicine, University of Alabama at Birmingham, Birmingham, AL, United States

**Keywords:** magnetic resonance imaging guided radiotherapy, positron emission tomography, stereoscopic imaging and surface guidance, cone beam computed tomography, generative image synthesis, Cherenkov radiation imaging, imaging innovations in proton therapy, advanced quantitative imaging

## Abstract

Radiation oncology is undergoing a transformative shift toward precision medicine through unprecedented advances in imaging technologies that enable increasingly personalized and adaptive cancer treatment. This comprehensive review synthesizes the underlying physical principles, current clinical applications, technical challenges, and quality assurance requirements across the complete spectrum of emerging imaging-guided radiation therapy approaches. We examine magnetic resonance-guided radiotherapy systems that enable daily soft-tissue visualization and online plan adaptation, positron emission tomography-guided platforms that allow real-time tracking of metabolically active tumor regions, advanced cone beam computed tomography systems supporting rapid adaptive workflows through artificial intelligence-enhanced image generation, and novel applications including Cherenkov radiation imaging and stereoscopic guidance with surface tracking. For proton therapy, we address innovations spanning dual-energy computed tomography, proton computed tomography, and *in-vivo* range verification that tackle fundamental range uncertainty limitations. In theranostics, we explore sophisticated quantitative imaging for personalized radiopharmaceutical dosimetry. Our analysis reveals that while these technologies converge to enable increasingly adaptive and biology-informed dose delivery, realizing their full clinical potential requires rigorous multicenter validation, standardized quality assurance protocols, integration of multi-omics with functional imaging, trustworthy automation with continuous performance monitoring, interoperable data pipelines, enhanced workforce training, and attention to equitable access across diverse patient populations. This integrated perspective provides a forward-looking framework to guide clinicians, medical physicists, and researchers in navigating the rapidly evolving landscape of precision radiotherapy while ensuring safe and effective implementation of these transformative technologies.

## 1 Introduction

Radiation oncology is experiencing a transformative shift toward precision medicine, driven by unprecedented advances in imaging technologies that enable increasingly personalized and adaptive cancer treatment. Traditional anatomical imaging approaches are rapidly being complemented and, in some cases, superseded by sophisticated modalities that integrate functional, molecular, and real-time biological information into treatment planning and delivery. This evolution represents a paradigm shift from static, one-size-fits-all radiation therapy toward dynamic, patient-specific approaches that can adapt to tumor biology, anatomical changes, and treatment response in real-time.

The emergence of magnetic resonance-guided radiotherapy (MRgRT) systems has enabled daily soft-tissue visualization and online plan adaptation, particularly transforming treatment of mobile targets, such as pancreatic and gastrointestinal malignancies. Simultaneously, the integration of positron emission tomography (PET) with linear accelerators has introduced biology-guided radiotherapy (BgRT), allowing real-time tracking of metabolically active tumor regions. Advanced cone beam computed tomography (CBCT) systems now support online adaptive workflows through rapid, high-quality imaging and artificial intelligence (AI)-enhanced CT generation. Novel applications, such as Cherenkov radiation imaging, stereoscopic guidance with surface tracking, and generative AI-based image synthesis, are further expanding the precision radiotherapy toolkit. In proton therapy, innovations spanning dual-energy CT, proton CT, and *in vivo* range verification address fundamental range uncertainty limitations, while theranostics applications demand sophisticated quantitative imaging for personalized radiopharmaceutical dosimetry.

This comprehensive review aims to provide a critical, integrated assessment of emerging and advanced imaging technologies that are reshaping precision radiotherapy. Unlike previous reviews that examine individual modalities in isolation, we synthesize the underlying physical principles, current clinical applications, technical challenges, and quality assurance requirements across the complete spectrum of imaging-guided radiation therapy (RT) approaches. Our analysis encompasses workflow considerations, automation potential, and standardization needs while highlighting how these technologies converge to enable increasingly adaptive, biology-informed dose delivery.

This article addresses a critical gap in the literature by providing the first comprehensive, cross-platform analysis of how diverse imaging modalities complement each other within modern precision RT workflows. We emphasize practical implementation challenges—including geometric accuracy requirements, quantitative imaging uncertainties, workflow optimization, and workforce training needs—that are essential for successful clinical translation but often underemphasized in technology-focused reviews. Furthermore, we outline a forward-looking framework that integrates multi-omics data with functional and anatomical imaging, supported by trustworthy AI automation and standardized quality assurance protocols. This integrated perspective is designed to guide clinicians, medical physicists, and researchers in navigating the rapidly evolving landscape of precision radiotherapy while ensuring safe, equitable, and effective implementation of these transformative technologies across diverse patient populations.

## 2 Magnetic resonance imaging-guided radiotherapy

Magnetic resonance imaging (MRI)-guided radiotherapy (MRgRT) allows for direct visualization of soft tissue anatomy during treatment and supports online plan adaptation. Two commercial systems have been deployed clinically. The ViewRay (Oakwood Village OH, USA) MRIdian platform combines a 0.35 T split magnet and was initially designed to utilize three cobalt sources, (1) but it has more recently incorporated a 6 MV linear accelerator (linac) (2). The Elekta (Stockholm, Sweden) Unity platform (3) couples a 1.5 T MRI with a 7 MV linac. Both systems incorporate MRI into the treatment room geometry but differ in field strength, system architecture, and clinical workflow. The MRIdian enables real-time beam gating using multiplanar cine imaging from a balanced steady-state free precession (bSSFP) sequence (4), while Unity employs high-resolution imaging and supports plan adaptation through structured workflows that include “Adapt to Position” and “Adapt to Shape” (5). These capabilities have enabled a shift toward online adaptive planning, where a new radiation treatment plan is created each day, accounting for inter-fractional changes in patient anatomy (6).

### 2.1 Technological challenges

The technological requirements for MRgRT are non-trivial. Radiation delivery hardware must function reliably in the presence of magnetic fields, and MRI performance must remain stable during beam-on conditions. Integration requires mitigation of mutual interference between linac and MR subsystems, attention to magnetic shielding, and spatial coordination of isocenters. In the case of MRIdian, the split-magnet design permits radiation beam access perpendicular to the B0 field, while modifications to RF shielding and gradient coil structure support simultaneous imaging and irradiation (2). The Unity platform adopts a different approach, positioning the linac outside the magnet bore and delivering radiation along the bore axis (3). These design choices reflect different approaches to managing electromagnetic interference, gradient performance, and beam access geometry (7). Differences in image quality arise largely not only due to differences in field strength (0.35 T for MRIdian vs. 1.5 T for Unity) but also from available sequences and system constraints related to simultaneous imaging and treatment. Sequences such as T2-weighted fast spin echo (FSE) and bSSFP have reduced acquisition times to fit within clinical workflows while preserving the image quality and spatial accuracy required for planning and guidance.

### 2.2 Clinical applications of MRgRT

In clinical practice, the most commonly used workflow on the MRIdian system involves daily acquisition of a bSSFP image, followed by manual or semi-automated recontouring, re-optimization of the treatment plan, and delivery with respiratory gating. When performed sequentially, the entire process takes approximately 45 min, though newer software versions (A3i) have enabled partial parallelization of workflow steps to reduce overall treatment time (8). Gating is based on direct visualization of the target or a surrogate structure in the cine image, with beam delivery suspended if the structure location exits a predefined boundary. On Unity, adaptation decisions are based on comparison between the reference and daily MR images. In the “Adapt to Position” workflow, the original plan is rigidly shifted, while in “Adapt to Shape,” a new plan is generated based on the recontouring of both target and critical structures (5). Unity now supports real-time gating, though this feature was only introduced after several years of clinical use. Its higher field strength enables improved soft tissue contrast and may also facilitate integration of functional imaging (9).

Pancreatic cancer has emerged as a primary disease site in which MRgRT has had a measurable clinical impact, particularly with the MRIdian system, which possesses a much longer gating functionality. Computed tomography (CT)-based planning for pancreatic tumors is limited by poor soft tissue visibility and motion of adjacent gastrointestinal organs, which limits how aggressively they can be treated (10). MR guidance permits direct visualization of the tumor and organs at risk (OARs) at the time of treatment, enabling tighter margins and more aggressive dose prescriptions (11). Initial retrospective series demonstrated the feasibility of delivering 50 Gy in five fractions with acceptable toxicity and local control (12). These results were tested prospectively in the SMART trial, which enrolled 136 patients with locally advanced or borderline resectable pancreatic cancer and treated them using the MRIdian system. The primary endpoint was gastrointestinal toxicity, which was observed in fewer than 5% of patients. Median overall survival exceeded 14 months, and 2-year survival was over 40% (13). These outcomes compare favorably to historical controls and have contributed to the growing interest in MRgRT for tumors in anatomically complex or mobile sites. The pancreas is not the only area of application. There is also emerging evidence in the liver, lung, and prostate, where online adaptation and motion mitigation may offer advantages in selected patients (14, 15). In CNS tumors, although not one of the common applications is present for MRgRT, daily MRI may support tighter margins and improved alignment with evolving anatomical changes during long courses of therapy (16). MRgRT enables treatment in anatomically complex or mobile sites where soft tissue visualization, motion management, or daily adaptation is required.

### 2.3 Geometric distortion in MRgRT

Geometric accuracy remains a foundational requirement for MRgRT. MRI is inherently prone to spatial distortion from static field inhomogeneities, gradient non-linearities, and magnetic susceptibility differences (17). These distortions can result in discrepancies between the true anatomical position and its appearance on MR images, particularly at the edges of the field of view and in the presence of air–tissue interfaces (18). The magnitude of distortion varies by sequence, field strength, and choice of imaging parameters. The MRIdian system exhibits relatively low distortion due to its low field strength (19, 20). The Unity system, owing to its higher field strength, exhibits greater distortion but benefits from more robust gradient performance and improved signal-to-noise ratio (21). The International Commission on Radiation Units and Measurements (ICRU) recommends a geometric accuracy of 2 mm or better for MRI used in radiotherapy planning and emphasizes the need for routine QA to assess image fidelity (22). These recommendations are particularly relevant for stereotactic treatments (23), with sharp-dose gradients, and for intracranial cases, where millimeter-level accuracy is clinically consequential. Institutions adopting MRgRT should incorporate distortion assessment into their commissioning protocols and account for potential residual uncertainties when defining margins.

### 2.4 Emerging MRI functional-guided RT

MRI enables the non-invasive interrogation of some biological functions *in vivo* (24–27). Incorporating these capabilities into MR-guided radiotherapy remains an area of active investigation and represents a potential future direction for adaptive treatment. Functional MRI techniques, such as diffusion-weighted imaging (28), dynamic contrast-enhanced MRI (29, 30), and MR spectroscopy (31), offer the possibility of quantifying tumor biology in real time. These approaches are of particular interest in adaptive strategies where the dose may be modulated based on early treatment response. Diffusion imaging has received the most attention due to its relatively short acquisition times and growing evidence of its correlation with cellular density and therapeutic response (32). Changes in apparent diffusion coefficient (ADC) during treatment have been proposed as an early biomarker of response and may support biologically driven adaptation (33). A recent clinical trial exemplifies this approach using mid-treatment ADC changes along with changes in perfusion to stratify patients with soft tissue sarcoma into dose escalation arms (34). These efforts are preliminary, and further validation is needed to establish the reproducibility and prognostic value of such biomarkers across disease sites and platforms. Nonetheless, the capacity of MRgRT to support quantitative imaging during the course of therapy positions it as a candidate platform for future biologically adaptive radiotherapy (35, 36). Challenges remain in sequence standardization, motion management during functional imaging, and integration with planning software, but these are areas of active investigation (37).

### 2.5 Summary

MR-guided radiotherapy has created new possibilities for daily adaptation, motion management, and soft tissue visualization. Its clinical use has expanded most rapidly in tumors where conventional image guidance has been limited and intra-fractional motion is substantial, particularly in pancreas and other gastrointestinal malignancies. MR-guided systems enable daily adaptation and motion management while offering a platform for future integration of biologically informed planning. As the field moves toward more precise and personalized therapy, MRgRT offers a platform that supports both current adaptation strategies and future functional imaging applications (38). Its full clinical impact will depend on continued technical refinement, validation of emerging biomarkers, and systematic incorporation into disease-specific treatment paradigms.

## 3 PET-guided RT: advancing precision in oncology

Positron emission tomography (PET) has revolutionized oncology by providing functional and molecular insights into tumor biology, complementing traditional anatomical imaging. The integration of PET with radiation therapy (RT) has led to the emergence of PET-guided RT, a sophisticated approach aimed at enhancing treatment precision, optimizing dose delivery, and improving patient outcomes (39, 40). This review explores the fundamental principles of PET imaging, its evolution, and diverse applications in radiation oncology, including target delineation, adaptive radiotherapy, and dose painting. It also delves into the development of integrated PET-Linac systems that enable real-time guidance. Furthermore, this addresses the current challenges and limitations in the clinical implementation of PET-guided RT, such as image quantification issues and logistical complexities, while highlighting promising future directions, including the role of artificial intelligence and novel radiotracers.

RT is a cornerstone of cancer treatment, aiming to deliver a precise dose of radiation to malignant cells while sparing surrounding healthy tissues. The evolution of RT has been driven by continuous advancements in imaging technologies, allowing for increasingly accurate tumor localization and treatment delivery. PET, a nuclear imaging technique, has emerged as a critical tool in oncology, offering unique functional insights by visualizing metabolic and molecular processes within the body. Unlike conventional imaging modalities, such as CT and MRI, which primarily provide anatomical information, PET can detect changes at the cellular level, potentially identifying disease in its earliest stages and assessing treatment response (41).

The integration of PET imaging into the RT workflow has paved the way for PET-guided RT, a paradigm shift toward more personalized and biologically informed cancer treatment. This review aims to provide a comprehensive overview of PET-guided RT, covering its underlying principles, clinical applications, technological advancements, current challenges, and prospects.

### 3.1 Principles of PET imaging and PET-guided radiotherapy

#### 3.1.1 Biophysics of PET

Imaging PET operates on the principle of detecting radiation emitted from radiopharmaceuticals (also known as radiotracers) injected intravenously into a patient. These radiotracers are molecules labeled with a small amount of radioactive material, designed to accumulate in specific tissues or bind to particular proteins, such as those found in tumors or areas of inflammation. The process involves positron emission, annihilation, coincidence detection, and image reconstruction.

#### 3.1.2 PET Radiotracers in oncology

The utility of PET in oncology is significantly enhanced by the availability of various radiotracers that target specific biological processes or cancer types. Fluorine-18 Fluorodeoxyglucose (^18^F FDG) remains the primary, FDA-cleared radiotracer for SCINTIX BgRT in lung and bone tumors (42). It enables real-time PET-based treatment adaptation using tumor metabolism as a dynamic fiducial marker. Gallium-68/Fluorine-18 Prostate-Specific Membrane Antigen (Ga-68 PSMA/F-18 PSMA) has been shown to enable PET-guided treatment planning for prostate cancer metastases, including bone lesions, within RefleXion^®^ 's BgRT workflow (43–45). It offers superior sensitivity for detecting recurrent disease and precise localization of metastatic lesions, even at low PSA levels. A brief BgRT tracer is summarized in Table 1, in addition to the tracers utilized in the clinic.

**Table 1:** A summary of the BgRT tracer and its utilization in the clinic.

**Tracer (Radiopharmaceutical)**	**Indication /Tumor types**	**Role in BgRT**
^18^F-FDG	Lung and bone tumors (primary/metastatic)	Standard tracer for real-time PET-guided BgRT (FDA-cleared for SCINTIX BgRT in lung and bone cancers)
68Ga-PSMA	Prostate cancer with bone metastases	Feasibility demonstrated for SCINTIX BgRT treatment in metastatic prostate cancer
^18^F-FES (FES = Fluoroestradiol)	ER+ breast cancer lesions	Pilot study comparing FES and FDG PET metrics for BgRT eligibility in breast cancer
64Cu-ATSM	Locally advanced rectal cancer	Studied for hypoxia-guided dose painting in BgRT-like planning (for Phase I feasibility planning only)
FAPI-based tracers (e.g., 68Ga-FAP-CHX, ^18^F-NOTA-FAPI, 68Ga-FAPI-JH04)	Various epithelial cancers	Promising for tumor microenvironment–guided dose targeting; ongoing dosimetry and biodistribution studies
Nectin-4/αvβ3-targeting tracers (e.g., 68Ga-N188, ^18^F-FAPI-RGD)	PD-L1/angiogenic tumors	Novel targets being tested via imaging trials; potential for BgRT guidance in immuno-oncology

### 3.2 Clinical applications of PET-guided radiotherapy

PET has become increasingly important in oncology, offering molecular-level visualization and quantification of tumor characteristics that extend beyond conventional morphologic imaging (7). These data enable (1) precise delineation of radiotherapy (RT) target volumes; (2) ongoing assessment of treatment response and effectiveness; (3) prediction of failure patterns by identifying sub-regions at high risk of recurrence; and (4) individualized dose adaptation, allowing escalation or de-escalation, where clinically warranted.

#### 3.2.1 Target delineation and treatment planning

PET imaging, often combined with CT (PET/CT) (46, 47) or MRI (PET/MRI) (48), provides comprehensive insights into tumor biology, improving diagnostic accuracy and enhancing patient positioning for RT. PET/CT has become a standard tool for cancer detection and staging, helping to identify tumors not visible on anatomical imaging and assessing tumor activity. PET/MRI, due to its high soft tissue contrast, offers unique advantages in T-staging of various cancers and is superior in finding lymph node and distant metastases compared to CT, MRI, and PET/CT.

#### 3.2.2 Adaptive radiotherapy (ART)

Adaptive radiotherapy (ART) is a refined approach that adjusts treatment plans to dynamic anatomical and physiological shifts within a patient's body during therapy. Through frequent imaging, including PET-guided ART (49, 50), it allows specialists to visualize changes like tumor shrinkage or organ movement, enabling precise modification of the radiation dose.

There are three levels of adaptation. (a) Offline ART: Involves adjusting treatment plans between sessions based on periodic imaging assessments. (b) Online ART: Modifies treatment plans immediately before delivery using on-couch imaging, with advanced software and AI facilitating rapid adjustments to daily anatomical variations. (c) Real-Time ART: Continuously adjusts treatment delivery based on real-time changes, creating a “living” picture for immediate detection of changes and ensuring optimal targeting. RefleXion^®^ X1 platform integrates PET imaging before and during treatment sessions, and it is the pioneer that can carry out the above-mentioned offline and real-time ART.

### 3.3 Integrated PET-linac systems

**Figure 1:**
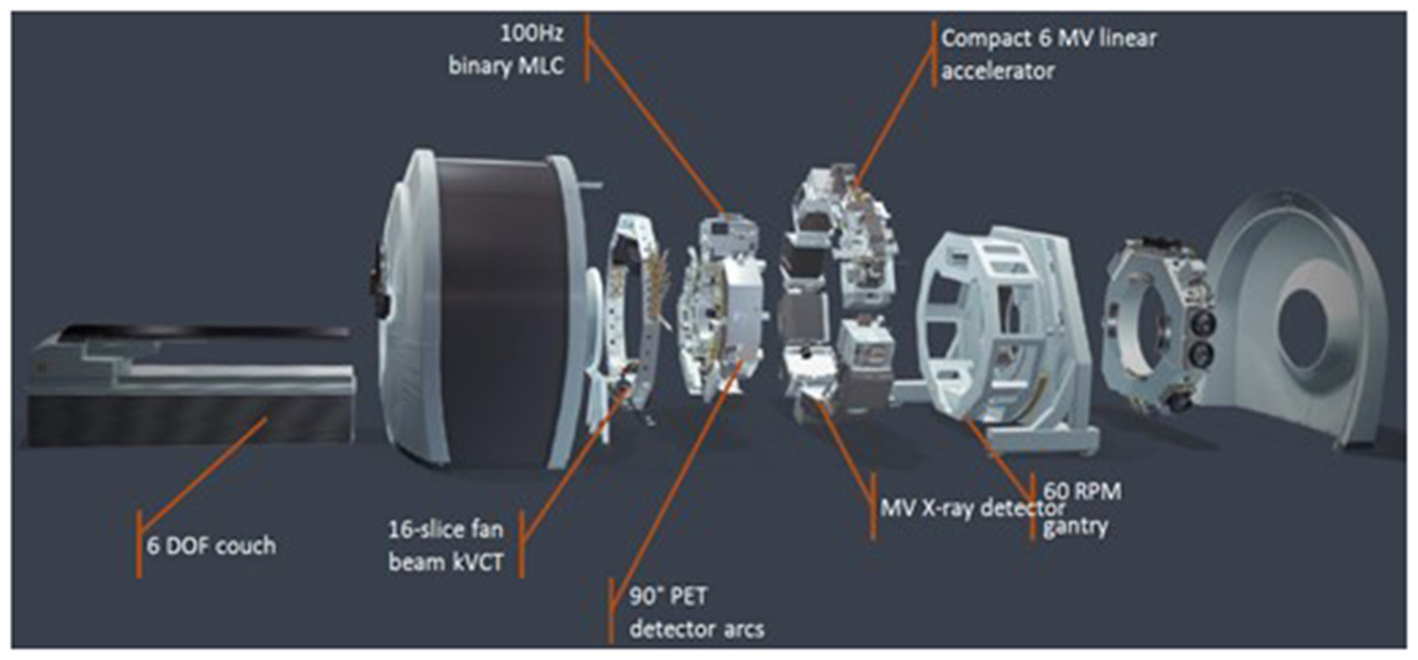
Major components: KVCT, PET detector arcs/MLC, MV, and 60 rpm gantry (Courtesy of RefleXion^®^ Medical).

The integration of PET detectors with linear accelerators represents a technological advancement in RT (51, 52). The RefleXion^®^ X1 system (53, 54), for example, is characterized by its split arc design, employing two 90° PET arcs to guide therapeutic radiation beams in real-time with sub-second latency. This system also incorporates an onboard fan-beam kVCT for anatomical capabilities, offering a hybrid platform for both biologic and anatomic guidance. The workflow for BgRT involves radiotracer injections and X1 PET scans as part of treatment planning, and immediately before and during each fraction for real-time guidance (55)_._ This real-time guidance allows for improved motion management for dose delivery accuracy (56, 57). Figure 1 shows the major components of RefleXion^®^ X1 PET/CT linac.

### 3.4 Challenges and limitations

#### 3.4.1 Image quality and quantification spatial resolution and noise

Despite the significant advancements, several challenges and limitations persist in the widespread clinical implementation of PET-guided RT.

Image Quality and Quantification Spatial Resolution and Noise: PET images often suffer from low spatial resolution and high noise characteristics, which can make accurate delineation of target regions problematic. The spatial resolution of PET systems is typically limited to around 5 mm.

Image Segmentation: Accurately segmenting tumors from blurred and noisy functional PET images is a difficult issue for PET-based treatment planning. While various segmentation approaches exist (e.g., thresholding, edge detection, and deep learning), their reliable performance on clinically relevant tasks requires objective, task-based evaluation. Inaccuracies can arise from variations in biological processes governing tracer uptake and physical/acquisition phenomena.

Quantitative Accuracy: Fundamental trade-offs between resolution and noise, along with challenges in scatter correction and attenuation correction, affect the quantitative accuracy of PET measurements.

#### 3.4.2 Specificity of radiopharmaceuticals

While radiotracers like 18F-FDG are highly sensitive to many cancer types, they are not always specific to malignant disease, as uptake can occur in other processes with increased glucose turnover, such as infection and inflammation. This can hamper the differentiation between inflammatory changes and neoplastic tissue, or between benign lesions and well-differentiated malignant lesions with low FDG avidity. Although more specific tracers like Ga-68 PSMA have been developed, sources of false positive or negative findings can still exist.

While radiotracers like ^18^F-FDG are highly sensitive to many cancer types, they are not always specific to malignant disease, as uptake can occur in other processes with increased glucose turnover, such as infection and inflammation. This can hamper the differentiation between inflammatory changes and neoplastic tissue, or between benign lesions and well-differentiated malignant lesions with low FDG avidity. Although more specific tracers like Ga-68 PSMA have been developed, sources of false positive or negative findings can still exist.

The involvement of radiopharmaceuticals in PET-guided radiotherapy and the short half-life of many radiopharmaceuticals, particularly ^18^F-FDG (110 min), necessitates that cyclotrons be located close to the radiation oncology department. The increased patient treatment time and complexity of the entire BgRT workflow require extra labor forces and seamless coordination among physicians, nurses, nuclear medicine technicians, therapists, and others. Regulation and guidelines, such as task group reports from the American Association of Physicists in Medicine (AAPM), are under development and improvement (58).

### 3.5 Future directions

The field of PET-guided RT is continuously evolving, with several promising avenues for future development:

- Novel radiotracers: Research is expanding to include available and novel tracers targeting tumor metabolism, hypoxia, vascularity, and proliferation, enabling more precise dose painting and adaptive strategies.
- Artificial Intelligence (AI) and Machine Learning (ML): In PET-guided adaptive radiotherapy, artificial intelligence and machine learning are being applied through several complementary approaches that directly address clinical bottlenecks. Deep learning models, such as 3D U-Net (59), V-Net (60), and residual networks, integrate PET's metabolic data with CT's anatomical details to generate accurate tumor and organ-at-risk contours, reducing the variability and time associated with manual delineation while overcoming challenges of physiologic uptake and heterogeneous tumor activity (61). Radiomics-based methods further enhance treatment personalization by extracting quantitative texture and wavelet features from PET scans to predict response and guide adaptation, including delta-radiomics analyses that track metabolic changes during treatment (62). Together, these AI tools aim to transform PET-based radiotherapy from a labor-intensive, subjective process into a standardized and adaptive workflow that supports real-time clinical decision-making.
- Multiomics Integration: Combining PET imaging with genomic, proteomic, and other omics data to provide more comprehensive biological insights for tailored treatment strategies.
- Polymetastatic Patient Treatment: The ambition to extend BgRT to polymetastatic patients in the future, potentially in conjunction with systemic therapy, represents a significant area of growth.
- Clinical Validation: Standardized segmentation protocols and prospective clinical trials are needed to validate clinical benefits and establish PET-guided RT in routine care.

### 3.6 Summary

PET-guided RT represents a significant leap forward in precision oncology, moving beyond anatomical targeting to incorporate real-time biological information for optimized treatment delivery. By leveraging the functional insights provided by PET imaging and advanced radiotracers, clinicians can achieve more accurate tumor delineation, implement adaptive treatment strategies, and explore dose painting techniques to personalize therapy. While challenges related to image quantification, radiotracer specificity, and logistical complexities remain, ongoing technological advancements, particularly in integrated PET-linac systems and the application of AI, are poised to overcome these hurdles. The continued evolution of PET-guided RT holds immense promise for improving disease control, minimizing toxicity to healthy tissues, and ultimately enhancing the quality of life for cancer patients.

## 4 Stereoscopic imaging and surface guidance techniques for central nervous system tumors

Stereoscopic imaging represents a paradigm shift in precision radiotherapy for central nervous system (CNS) tumors, enabling submillimeter positioning accuracy through dual oblique X-ray imaging systems. Unlike conventional single-plane imaging, stereoscopic techniques provide target localization by triangulating anatomical landmarks from two simultaneously acquired oblique projections. Commercially available systems currently include ExacTrac (Brainlab, Munich, Germany), SyncTraX (Shimadzu, Kyoto, Japan), and CyberKnife (Accuray, Sunnyvale, CA, USA) (63). Among these systems, ExacTrac is the most used as an add-on imaging system to a medical linear accelerator. Stereoscopic imaging has become particularly crucial for intracranial stereotactic radiosurgery (SRS) and spinal stereotactic body RT (SBRT), where high-dose single or hypo-fractionated treatments demand exceptional geometric precision to accurately deliver therapeutic doses to lesions while sparing adjacent critical structures (64–66).

### 4.1 Technical principles and system architecture

The fundamental architecture of the ExacTrac imaging system consists of two kilovoltage (kV) X-ray sources recessed into the treatment room floor and the corresponding ceiling-mounted amorphous silicon flat panel detectors positioned in an oblique configuration (67). Other stereoscopic imaging systems use some variation of this geometric arrangement. The stereoscopic imaging system is calibrated such that its imaging coordinates are accurately matched to the linac's coordinates. This oblique geometric arrangement enables the acquisition of two instantaneous stereoscopic images without source-detector repositioning, facilitating continuous monitoring throughout treatment delivery, even at non-coplanar patient couch angles where conventional linac-based onboard imaging systems face geometric limitations and the risk of gantry-couch collision.

The dual X-ray generator configuration produces high-resolution stereoscopic images with adjustable kilovoltage, and the tube current parameters are optimized for different anatomical structures and imaging requirements. Typically, 90 kVp and 10 mAs are used for cranial applications, and 120 kVp and 20 mAs are used for spinal applications, though these parameters can be adjusted according to patient size and anatomy. Advancements such as higher heat capacity X-ray tubes support more frequent automated imaging sequences, while enhanced soft tissue contrast and improved readout speeds minimize motion blurring artifacts. Modern systems incorporate larger imaging panels that visualize extended anatomical regions, improving image interpretation and anatomical orientation. Advanced image fusion algorithms match acquired stereoscopic projections with digitally reconstructed radiographs (DRRs) from planning CT datasets, enabling precise six-degrees-of-freedom (6DOF) patient positioning corrections (67).

### 4.2 Evolution from infrared tracking to thermal surface guidance

Early ExacTrac systems utilized infrared-reflective spheres mounted on patient positioning arrays: a cranial matrix integrated with a face mask or reflective markers attached directly to the mask for intracranial treatments and a reference U-shaped array mounted on the couch sidebar for extracranial applications. Reflective markers can also be directly attached to other immobilization devices or the patient's skin. While effective, this approach required rigid body assumptions and was limited to tracking discrete marker points rather than comprehensive patient surface geometry. The accuracy of the infrared tracking depends on the quality and stability of the reflective markers, which fade with time. The positioning array served merely as a surrogate for patient motion, and its movement could not accurately reflect the patient's actual anatomical displacement. Additionally, the mechanical connection of the U-shaped array to the couch sidebar was inherently unstable, with potential for disconnection or displacement during treatment delivery, which could compromise motion monitoring reliability.

The advancement to the ExacTrac Dynamic system implements markerless surface tracking through 4D thermal camera technology. This system correlates patient heat signatures with reconstructed three-dimensional surface structures, acquiring approximately 300,000 surface points matched to thermal signatures. Thermal surface guidance provides comprehensive patient surface monitoring that eliminates the need for positioning arrays while maintaining submillimeter accuracy.

### 4.3 Clinical implementation in CNS stereotactic treatments

#### 4.3.1 Initial patient setup and positioning

Patient positioning begins with the placement of the patient on the treatment couch using appropriate immobilization devices. Surface/thermal imaging provides rough initial alignment to the planning CT-generated patient surface contour. After initial stereoscopic X-ray images are acquired, suggested shifts are calculated automatically by matching the X-ray images to the reference DRRs generated from the CT simulation dataset. The calculated shifts will be sent to the 6DOF robotic couch to achieve optimal patient alignment. When positional deviations exceed system correction capabilities, manual patient repositioning is required before re-imaging. This iterative process continues until all translational and rotational parameters fall within preset tolerance thresholds, which are institution-specific and may vary based on treatment site and clinical experience. For example, tolerances of 0.5 mm/0.5° are commonly used for cranial applications, while spinal treatments may require larger tolerances (e.g., 0.7 mm/0.8°) due to the inherent challenges of reproducing exact spinal curvature and the difficulty of achieving submillimeter precision for vertebral positioning.

#### 4.3.2 Additional verification of positioning accuracy with onboard imagers

Images acquired using the linac onboard imaging system can serve as secondary confirmation of patient positioning accuracy. For cranial cases, a kV/kV image pair acquired at couch angles near zero degrees can be used since it provides optimal anatomical visualization for skull-based registration. Spinal treatments utilize a more comprehensive verification imaging approach, incorporating kV/MV image pairs alongside cone-beam computed tomography (CBCT) to improve vertebral anatomy visualization and account for potential differences in spinal curvature between simulation and treatment setup. This secondary imaging confirmation is particularly crucial for spinal treatments, where vertebral bodies may appear similar on stereoscopic projections, and can reduce the risk of patient setup on incorrect vertebral levels. CBCT imaging allows careful review of the spinal cord canal position relative to the target, providing critical safety verification. In addition, CBCT or kV/MV imaging can also help visualize anatomical changes and patient weight loss/gain. While many centers do not routinely use secondary imaging with ExacTrac, this additional verification step can prevent targeting errors and enhance treatment safety. Deviations detected by onboard imaging are not used to adjust patient positioning. Usually, for CNS cases, only ExacTrac stereoscopic X-ray images are used to calculate and apply couch shifts for patient repositioning. However, this practice is institution-specific and may vary based on treatment site and clinical experience.

#### 4.3.3 Multi-disciplinary image review process

All acquired images undergo systematic review by qualified medical physicists and radiation oncologists to ensure treatment accuracy and patient safety (64, 65). This verification process includes assessment of daily anatomical variation and image registration quality, evaluation of target positioning accuracy, and confirmation of critical structure avoidance. The radiation oncologist provides final approval through the Record and Verify system, while the medical physicist independently verifies all beam parameters and delivery settings before treatment initiation.

#### 4.3.4 Pre-beam and intra-fractional verification protocol

**Figure 2:**
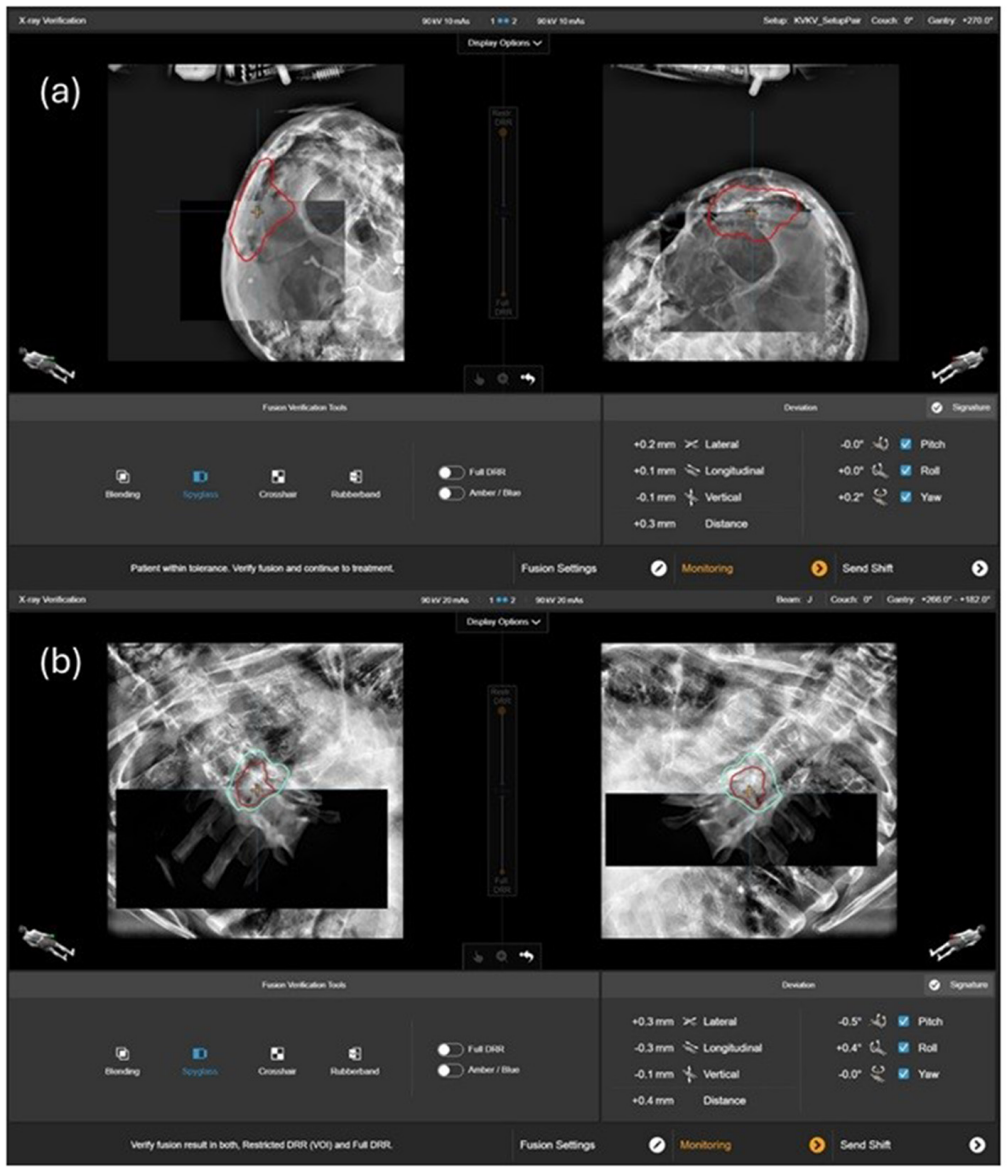
X-ray verification of stereoscopic images for a cranial **(a)** and a spinal **(b)** case.

Prior to the delivery of each treatment beam, verification of stereoscopic images is acquired to confirm the maintained patient alignment. When deviations exceed tolerance thresholds but remain within treatment system correction capabilities, calculated shifts are applied, followed by acquisition of confirmatory stereoscopic images. Only after verifying that all positioning parameters are within preset tolerances can treatment beam delivery start. Once positioning verification is achieved, new baseline thermal surface images are created using the 4D thermal camera system, establishing the reference patient surface geometry for subsequent motion monitoring during the selected treatment beam delivery. Figures 2a, b illustrate the verification of stereoscopic images for a cranial and a spinal case, respectively.

#### 4.3.5 Real-time motion monitoring during treatment

**Figure 3:**
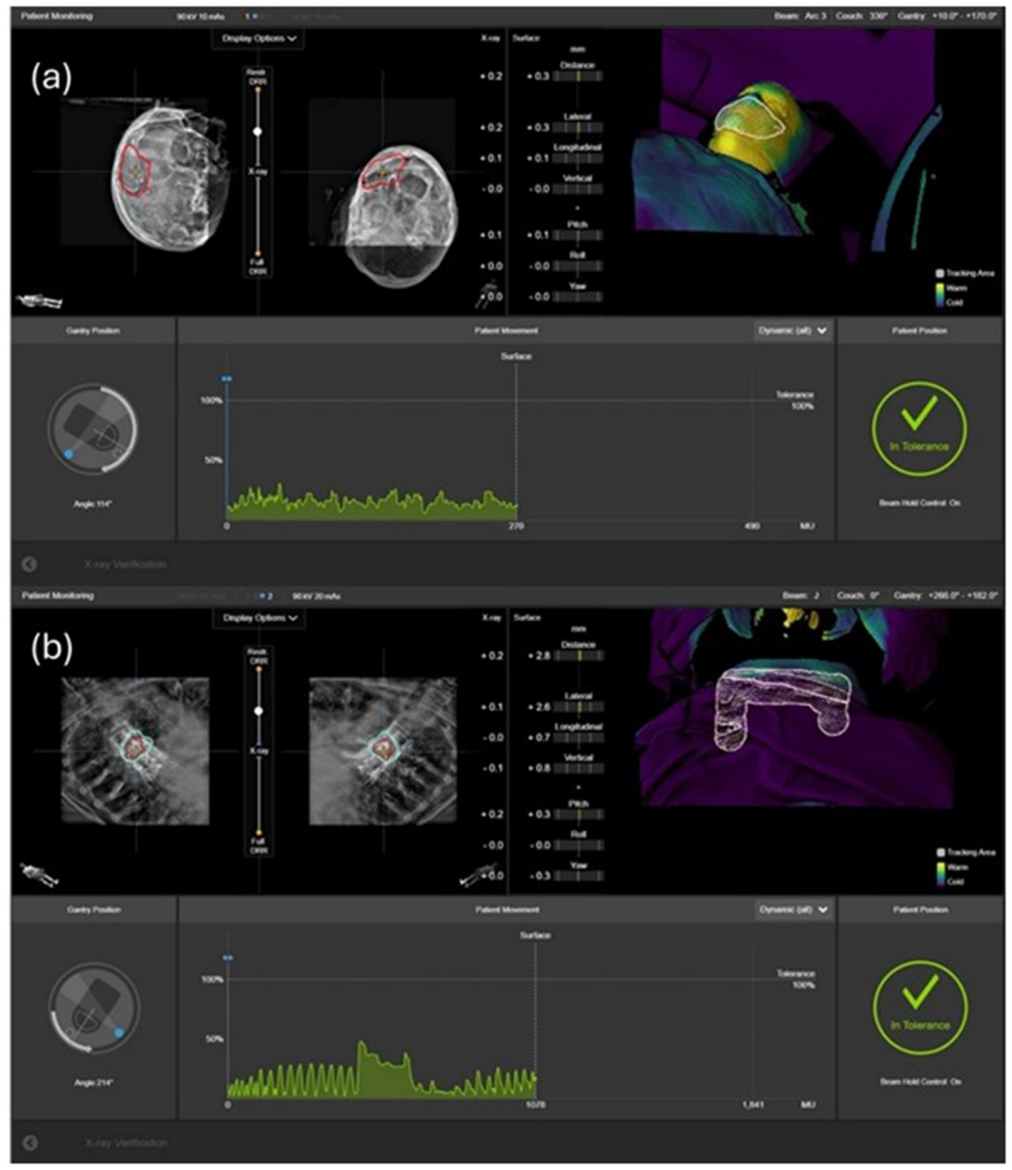
Real-time surface motion tracking alongside the pre-beam verification stereoscopic X-ray images for a cranial **(a)** and a spinal **(b)** case.

Throughout the treatment, continuous thermal surface tracking monitors patient motion in real time. The user defines specific regions of interest for surface tracking based on treatment site and clinical requirements. When patient motion exceeds preset tolerances within these monitored regions, automatic beam-hold functionality is immediately triggered to interrupt the treatment delivery. Surface tracking tolerances of 2.0 mm/2.0° are commonly used for cranial applications. However, for spinal treatments, the threshold may be relaxed since surface tracking is affected by respiratory motion, while the target vertebral structures themselves do not move with respiration. This respiratory artifact may require larger motion tolerances (>1 cm) before beam gating is triggered, limiting the clinical utility of surface monitoring for spinal cases. ExacTrac systems also allow automated X-ray triggering during treatment based on predefined gantry angles or monitor unit intervals, although this feature may not be utilized in all clinical scenarios due to practical considerations, such as limited arc ranges used for certain spine treatments. This continuous monitoring capability ensures maintained positioning accuracy throughout the entire treatment fraction, particularly crucial for lengthy stereotactic procedures where patient comfort and positioning stability may become challenging. Figures 3a, b illustrate the real-time surface motion tracking alongside the pre-beam verification stereoscopic X-ray images for a cranial and a spinal case, respectively.

### 4.4 Current limitations and future directions

Despite significant advances, stereoscopic imaging maintains inherent limitations as a projection-based technique. Two-dimensional projections may obscure anatomical details compared to volumetric imaging modalities, and the rigid body assumption underlying motion tracking may not capture subtle non-rigid patient movements. Thermal surface tracking may not accurately represent the motion of internal organs and can be affected by environmental factors, such as room temperature variations, air conditioning drafts, patient perspiration, and variations in patient skin temperature, which may alter thermal signatures and compromise tracking accuracy. Patient comfort considerations, particularly with tight-fitting immobilization masks, remain challenging for extended treatment sessions, although different types of masks (basic, open-face, and stereotactic) are available to meet different clinical needs.

Future developments might focus on enhanced thermal surface tracking algorithms that are more robust to environmental and physiological variations, improved soft tissue contrast capabilities, and integration with X-ray and/or magnetic resonance-guided volumetric imaging platforms. Advanced motion prediction algorithms and artificial intelligence-enhanced image fusion represent promising avenues for further improving positioning accuracy and workflow efficiency.

### 4.5 Summary

In summary, stereoscopic imaging and surface guidance techniques have fundamentally transformed precision radiotherapy for CNS tumors, enabling submillimeter accuracy essential for safe dose escalation in SRS/SBRT applications. The evolution from purely X-ray-based imaging systems to hybrid thermal-surface guidance platforms demonstrates continued technological advancement toward optimal patient positioning and motion management. These innovations directly support the clinical goal of maximizing tumor control while minimizing normal tissue toxicity, particularly crucial for treating lesions adjacent to critical neurological structures.

## 5 Online adaptive radiotherapy using Ethos

**Figure 4:**
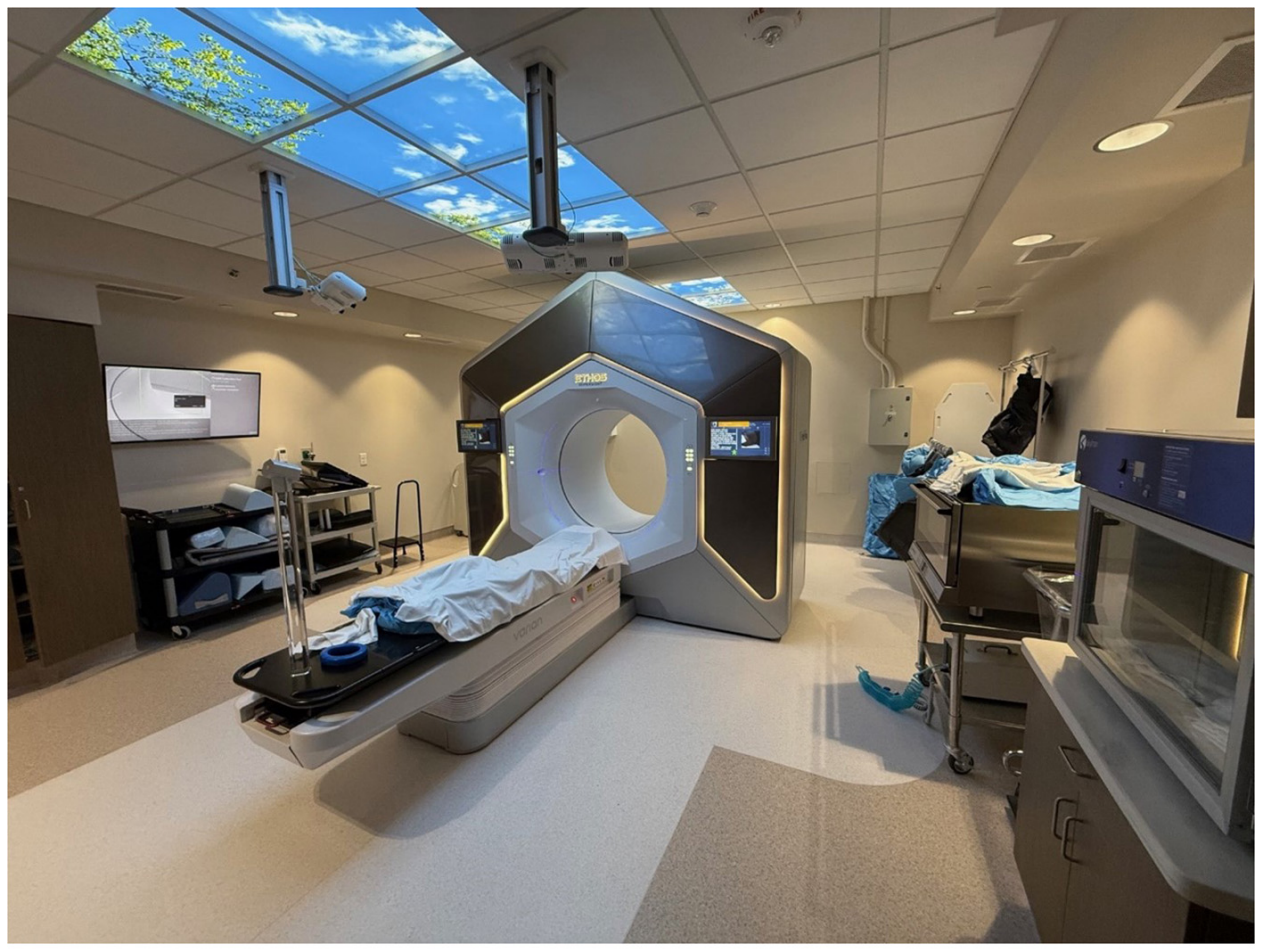
The all-in-one Ethos LINAC system, which can be used for CT-simulation, CBCT imaging, AI contours, GPU-based planning, surface guided imaging, QA, and treatment delivery.

The Ethos linear accelerator (Figure 4) is an online adaptive radiotherapy (OART) system with a ring-shaped gantry and an AI platform. As the FDA-cleared CBCT-guided OART device, Ethos enables high-quality, fast CBCT acquisition and on-couch treatment planning focused on patients' daily anatomical changes.

Ethos utilizes a single energy of 6 MV flattening filter free (FFF) beam and features a dual-layer multi-leaf collimator (MLC) design with staggered leaves, giving an effective 5 mm MLC thickness. The dosimetric leaf gap (DLG) is in the range of tenths of a millimeter, and the leaf transmission coefficient is around 0.01%, which is much smaller than the single-layer MLC design in a conventional C-arm linac. The maximum field size is 28 cm with the full MLC travel range of 28 cm. The bore size is 1 m in diameter, and the couch has three degrees of freedom (DOF). The closed bore and compact design allow for four revolutions per minute (RPM), enabling fast treatment and minimal collision risk (68). Compared to the conventional C-arm linac, Ethos has no field light, no optical distance indicator (ODI), and no laser marking at the treatment isocenter. Instead, it relies on external lasers for patient alignment and automated shifts in the software. Compared to conventional kV CBCT imaging, Ethos HyperSight provides metal artifact reduction, more accurate HU values, fast acquisition, and superior imaging quality with an extra-large kV imager (~70 cm) and a high-precision iterative CBCT reconstruction algorithm.

Furthermore, Ethos has a dedicated treatment planning system with a pre-configured beam model, an AI-driven automatic contouring, and a plan optimization algorithm called the intelligent optimization engine (IOE) (69). Some Ethos linacs are also equipped with a surface-guided imaging system for motion management and a Mobius quality assurance (QA) system for gamma analysis. All those features in the Ethos platform enable the efficient and accurate on-couch patient adaptive radiotherapy workflows.

### 5.1 Advanced imaging technology of Ethos OART

In traditional adaptive workflows, such as head and neck re-planning, subsequent CT is acquired after noticeable anatomical changes are observed in the daily CBCT image. Different treatment plans based on patient anatomy changes are created when patients are off-the-couch. Subsequently, daily CBCT images are obtained prior to treatment delivery to verify patient positioning for new plans. This is called image-guided radiotherapy (IGRT) or offline/off-the-couch adaptive radiotherapy. In contrast, Ethos online adaptive process starts with the patient's daily CBCT imaging to visualize anatomy change, followed by recontouring of organs at risk (OAR) and targets, and eventually planning optimization based on that day's patient anatomy while the patient is on the couch. The fundamental difference between the Ethos and traditional RT workflows lies in the timing of CT acquisition for treatment planning. The Ethos system generates adaptive plans based on daily CT scans obtained on each treatment day, whereas traditional adaptive RT relies on CT scans acquired weeks before new adaptive treatment begins. Theoretically, this allows for better OAR sparing and potential target dose escalation than the non-adaptive workflow because a new plan is created every day based on the evolving spatial relationship between tumor and normal tissue. Many body sites can benefit from OART, such as the male and female pelvic region (70–72), the upper abdomen region (73), breast cancer (74, 75), and lung cancer (76) due to variable organ volume or the daily motion. CBCT adaptive therapy is also useful for areas of anticipated weight loss, such as the head and neck (77). A recent study demonstrated that compared to traditional adaptive workflows, the Ethos OART system enables feasible daily adaptive treatments with reduced margins while enhancing target coverage and reducing OAR doses by up to 12 Gy for head and neck patients with oropharynx and larynx cancers (78).

**Figure 5:**
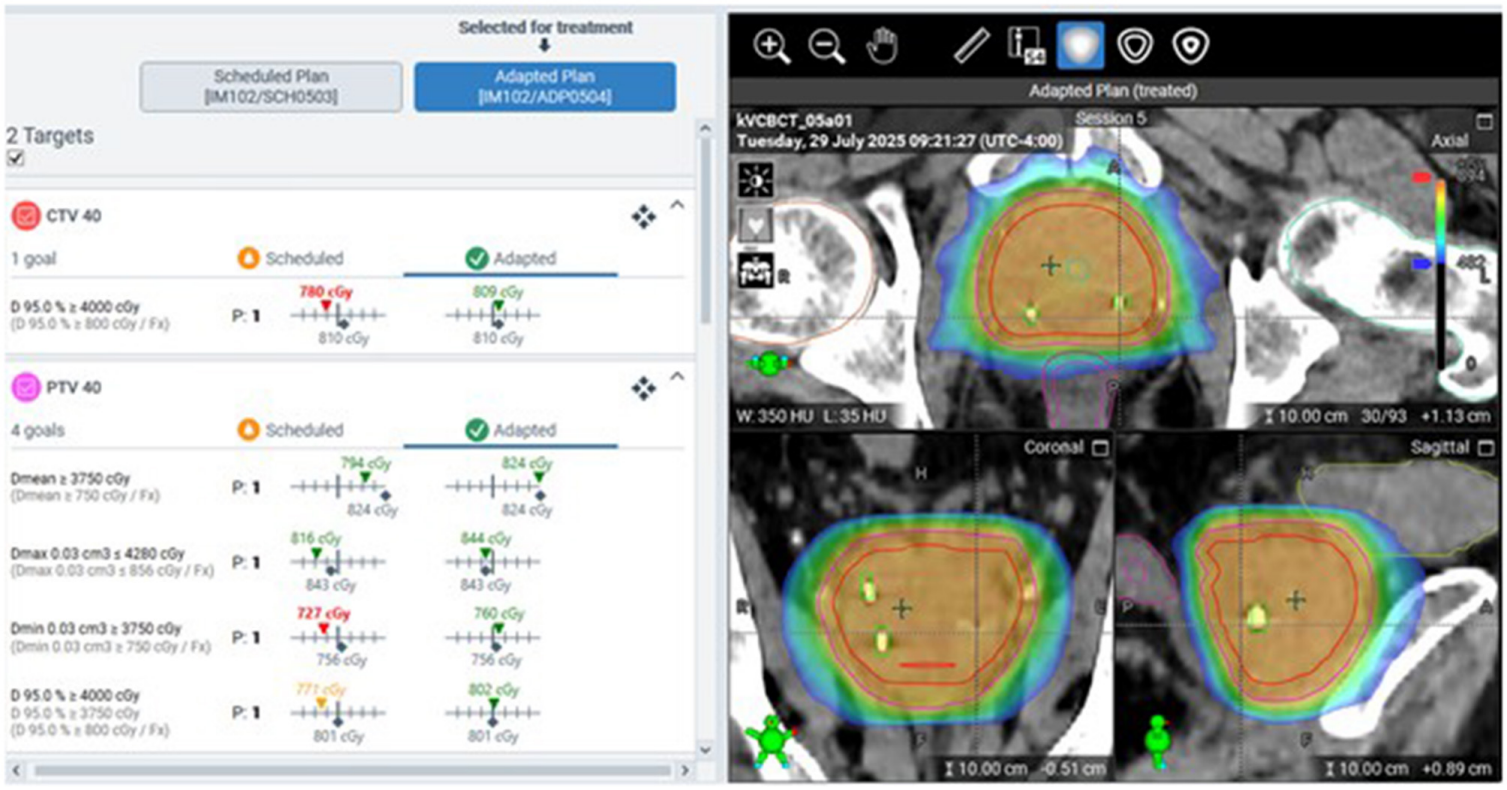
The adaptive plan is selected for treatment based on that day's CBCT. Fiducial markers are visible and used as the landmark for positioning verification during the on-couch adaptive process.

The HyperSight CBCT system has enhanced hardware and software components that contribute to improved scan quality and contour accuracy. The panel utilizes cesium iodide (CsI) scintillator material for higher conversion efficiency and fast readout. Compared to the prior generation of imaging panel, the HyperSight system has twice the active detector area (86 cm × 43 cm) with no lateral offset. This allows for a full-fan trajectory to be used, enabling an image acquisition time of 6 s. This faster scan time has been shown to provide reduced motion artifacts on the planning image (79). Since a plan is created based on daily contours, visualization of targets and OARs is critical to optimizing, calculating, and delivering the plan accurately. Prior to the introduction of HyperSight CBCT, the plan was calculated on a synthetic CT that mapped Hounsfield units (HU) from the simulation CT to the daily CBCT with deformable image registration. With the introduction of HyperSight on Ethos version 2.0, plans may be calculated directly on CBCT images (79). Reconstruction of CBCT images can be performed with the analytical Feldkamp-Davis-Kress algorithm or the iterative CBCT algorithm (iCBCT). An improved metal artifact reduction reconstruction algorithm, iCBCT Acuros MAR, is also included as a reconstruction mode for kV CBCT. Studies have shown that HyperSight CBCT image quality and HU accuracy are comparable to those of CT simulation images, suggesting the utility of the image data for direct dose calculation in adaptive workflows (80). Figure 5 demonstrates a CBCT-based prostate SBRT, where the adapted plan is selected over the scheduled plan for treatment because the target coverage is superior based on the specific bladder and rectal filling prior to treatment on that day.

One promise of direct dose calculation on CBCT is the use of the Ethos linac for simulation-plan-treatment workflows. This CT-simulation free workflow has been suggested for abdominal (81) and spine SBRT (82). By utilizing library plans or diagnostic images or even phantom plans as a “pre-plan,” we can acquire the patient's CBCT image and create an adaptive plan on the day of treatment. Therefore, there is a reduction in planning time, with some sessions involving only a single visit (83).

### 5.2 Planning and workflow considerations for online adaptive radiotherapy

Although most patients theoretically can benefit from adaptive radiotherapy, judicious use of planning and machine time resources is important. Adaptive treatments require extended time on the machine to allow for contour generation and plan review, leading to potential patient discomfort and movement. In addition, there are additional planning considerations in the Ethos system that require additional dosimetry or physics FTE (84). Workflows vary by clinic, with some having dosimetrists in a traditional role and others utilizing physicists for all Ethos planning (85). During planning, physics and dosimetry must consider visualization of the anatomy at the machine, accuracy of reference targets and structures with their derivations, and the robustness of planning goals to changes in daily patient anatomy. Planning images from simulation should ideally be free of contrast to avoid any issues with synthetic CT (86) or image registration and large enough to cover the anatomy of interest, but small enough for efficient optimization during planning. The Ethos system utilizes daily auto-contouring of patient anatomy to help aid in on-session planning speed, so accurate delineation of these organs at the time of initial planning is important for accurate deformable image registration and target delineation at the time of treatment.

The Ethos intelligent optimization engine (IOE) translates clinical goals input by the planner into planning optimization objectives in a piecewise continuous “quality” function (87). It then iterates the quality function on a priority-quality plane until a goal point is met and does not contribute to lower priority functions. In practice, clinical goals are grouped into priorities, the order of which influences this optimization process greatly. Due to this, the planning goals and their order need to be carefully considered, both on the planning image from simulation CT and with foresight on potential anatomical changes on the CBCT. With robust planning templates, online adaptive radiotherapy has been shown to have dosimetric benefits in several sites, such as breast (75), prostate (88), and lung (80). For example, it has been reported that the adaptive plan was the preference in 95% of fractions for prostate radiotherapy. Online adaptive radiotherapy may allow us to reduce the target margin while maintaining the tumor coverage and sparing critical organs nearby (88).

### 5.3 Summary

In summary, the high-quality CBCT imaging-guided online adaptive radiotherapy represents a unique opportunity for delivering customized plans based on daily patient anatomy. It uses high-performance kV imaging to visualize a patient's daily changing relationship between tumor and OAR, which is integrated with efficient contouring, intelligent optimization, and precise dose calculation. Therefore, kV CBCT-based online adaptive radiotherapy has great clinical potential for dose escalation in the tumor to enhance the local control, while sparing the critical structures or lowering the toxicity to OAR with reduced target margins.

## 6 Image synthesis in RT

Generative deep-learning-based image synthesis is an increasingly active area of research in radiation oncology. These techniques can create one imaging modality from another, offering new ways to streamline clinical workflows. CT remains the standard for simulation and treatment planning (89). CT volumes are reconstructed by inverting measurements of the linear attenuation coefficient (μ) collected at multiple projection angles with the Radon transform (90). As voxel values map directly to electron density, CT is indispensable for accurate dose calculation.

Other modalities, such as MRI, PET, and ultrasound, provide complementary information that can guide accurate dose delivery. However, acquiring these additional scans is often time-consuming, costly, or—under some circumstances—simply impractical for clinicians and patients. Recent advances in deep learning mitigate these barriers by enabling high-quality cross-modal image synthesis, thereby reducing the need for multiple acquisitions and opening new avenues for truly personalized RT.

### 6.1 Deep learning networks in medical images

Deep learning (DL)—a branch of machine learning built on multi-layered artificial neural networks—now underpins many techniques for generating synthetic images in RT. Recent review articles (91–95) survey the principal DL architectures applied in this field, with convolutional neural networks (CNNs), generative adversarial networks (GANs), and diffusion-based models emerging as the most widely used.

**Figure 6:**
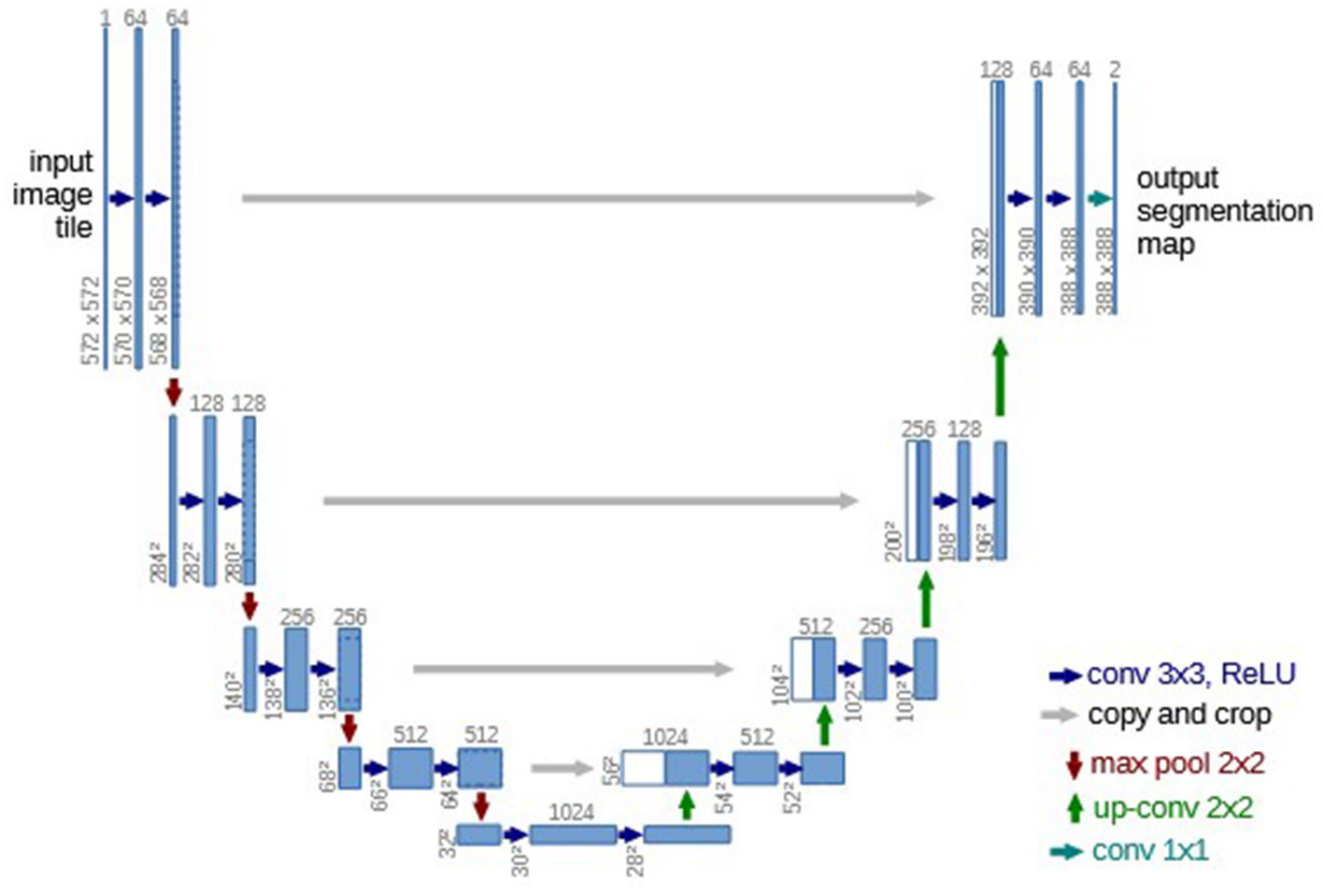
Architecture of the U-Net. Reproduced with permission from Ronneberger et al. (59).

CNNs are a class of deep-learning models inspired by the hierarchical organization of neurons in the human visual cortex (96–98). Purpose-built for grid-like data, they have become ubiquitous in medical imaging applications (99–102). Each convolutional layer deploys a bank of learnable filters that scan the input, capturing local patterns—such as edges and textures—while sharing parameters across the field of view to curb model complexity and ensure translation invariance. Stacking multiple convolutional layers with non-linear activation yields progressively abstract, hierarchical feature representations (103–105). Pooling (106) and other down-sampling operations (107) further condense contextual information, whereas random dropout (108) regularizes the network and mitigates overfitting. By learning features directly from data rather than relying on hand-crafted descriptors, CNNs have become the backbone of image analysis and synthesis tasks in RT. One of the most well-known CNN models is the U-shaped net (U-Net) proposed by Ronneberger et al. (59) (Figure 6). One important modification of the U-Net is direct skip connections between the encoder and the decoder. The U-Net does not have any fully connected layers. Instead, it only uses the valid part of each convolution, which allows the network to propagate context information to the up-sampling layers.

GAN was introduced by Goodfellow et al. (109). Compared to the image generated by the CNNs, it further improves the image quality. GANs learn to synthesize realistic data through a game-theoretic contest between two neural networks: a generator (G) and a discriminator (D). The generator tries to produce realistic images that resemble the real training distribution, while the discriminator simultaneously learns to distinguish generated images from genuine ones. During training, each network improves in response to the other's progress: the generator refines its outputs to fool the discriminator, and the discriminator improves its ability to detect fake images, creating a dynamic “adversarial” loop that gradually drives the generator toward high-fidelity outputs. This framework has enabled breakthroughs in photorealistic image synthesis. Derivative networks, such as Conditional GANs (110, 111) , CycleGAN (112, 113), and StyleGAN (114, 115), extend the idea to guided generation, unpaired domain translation, and finely controllable synthesis, making GANs one of the most versatile and influential tools in modern machine learning.

Another category of generative model is the diffusion model, which was first introduced in 2015 by Sohl-Dickstein et al. (116). Diffusion models generate images by gradually contaminating training data with Gaussian noise and then learning to reverse this process iteratively. Instead of learning the image itself, the model is trained to learn the contaminated noise, which effectively denoises the dataset into realistic samples. Their iterative nature yields high-fidelity detail and inherent diversity, making them more robust to noise contamination in the training dataset. Recent studies show that denoising diffusion probabilistic models can synthesize 3-D MRI and CT volumes with realistic image quality (117, 118). Emerging “foundation” approaches such as MedDiff-FM aim to unify multiple tasks—synthesis, reconstruction, and denoising—within a single large diffusion backbone, pointing toward versatile, privacy-preserving generative pipelines across modalities (119). These advances collectively position diffusion models as a core engine for safe, scalable medical image synthesis.

### 6.2 Application of image synthesis in RT

Substantial progress has been achieved in image synthesis applications for RT. Notable examples include MRI to synthetic CT (sCT) conversion (120–125) and synthetic MRI (sMRI) (126–134), synthetic PET (sPET) (127, 135, 136), and CBCT to sCT conversion (137, 138).

In recent years, interest in MRI-guided RT has grown substantially within the radiation oncology community. Compared with CT, MRI offers superior soft-tissue contrast and exposes patients to no additional ionizing radiation. This advantage allows more precise delineation of tumors and organs at risk, such as the bowel and optic nerves. MRI signal intensity, however, depends on sequence-specific parameters—e.g., repetition time (TR), echo time (TE), flip angle, and inversion time (TI) (139)—and therefore lacks a direct, one-to-one correlation with electron density. As a result, MRI alone cannot support accurate dose calculation. To address this limitation, generative AI models are now used to synthesize sCT images from MRI data, making MRI-only treatment planning feasible (140, 141). Several studies have quantified the dosimetric differences between sCT and the reference planning CT (125, 142–145). In photon therapy, the mean dose deviation is approximately 1% (142). By contrast, proton therapy is more sensitive: reported proton-beam range shifts reach 5.6 mm in liver cancer (125) and 7.5 mm in prostate cancer (145), which can translate into clinically significant dose errors.

Synthetic MRI leverages advanced machine learning models—most commonly CNNs (96), GANs (109), or diffusion models (116)—to rapidly generate high fidelity MR-like images from either undersampled k-space data or alternative inputs such as CT, quantitative maps, or single contrast scans (127, 146). By learning the complex, non-linear relationship between tissue properties and MR signal formation, these models can synthesize multiple contrasts (e.g., T1, T2, or FLAIR weighted images) (147) in a single inference step, standardize intensity across patients, and even predict quantitative relaxation parameters (148). The result is a dramatic reduction in acquisition time and patient motion artifacts, more consistent image quality, and the potential to extend MRI-level soft tissue visualization to scenarios where full MRI is impractical—such as RT workflows that rely primarily on CT (149). As the techniques mature, synthetic MRI is poised to streamline imaging protocols, lower costs, and enable new precision medicine applications ranging from adaptive treatment planning to longitudinal disease monitoring.

PET is already a powerful tool in radiation oncology, which provides functional information about the metabolism of the tissues, but practical and technical barriers keep it from being used whenever it would add value. Synthetic PET imaging is driven by deep learning generators that learn to translate structural or low-count inputs into realistic tracer uptake maps (150). The field began with 3-D U-Nets that capture the global, non-linear correlation between whole-brain MRI volumes and FDG activity (151) and quickly moved to conditional GANs—such as the globally and locally aware GLA GAN (152)—which combine adversarial, pixel-wise, and structural similarity index measure (SSIM) losses so both coarse context and fine lesion details are recovered. Further refinements include frequency-aware U-Nets (153) that process low- and high-frequency bands separately to sharpen edges and textures, and bidirectional or reversible GANs (154, 155) that embed PET semantics in a shared latent space to enforce cycle consistency and boost perceptual fidelity. More recent architectures add Transformer attention to fuse multi-modal MRI/PET cues and model long-range dependencies (156) or adopt diffusion models that iteratively denoise random noise under MRI or textual guidance to yield high-fidelity standardized uptake values (SUVs) (157). These innovations underpin applications such as synthesizing full dose scans from low dose PET or from MRI alone (127, 158, 159), mitigating noise while preserving quantitative accuracy and thereby reducing radiation burden for patients.

CBCT acquired on the treatment machine employs a cone-shaped beam and a flat panel detector; scatter from the whole patient therefore overwhelms the signal, creating streaking, cupping, and other artifacts that corrupt HU (160) accuracy and compromise dose calculation (161). Deep learning pipelines now correct these limitations by translating CBCT into synthetic CT (sCT) volumes with calibrated HUs. Two complementary strategies dominate: projection domain correction (162), in which CNN or GAN models clean hundreds of 2 D x ray projections before reconstruction—leveraging the rich (>300) projection set to converge quickly and bypass many image domain artifacts—and image domain translation (163), where architectures such as U-Net (1), CycleGAN, (112, 113) or attention GAN (137) act directly on the reconstructed CBCT to recover CT-like contrast and bone detail. Projection domain networks can even be trained on non-anthropomorphic phantom projections to learn scatter patterns, enhancing generalizability, while many image domain studies rigidly register CBCT and planning CT to minimize geometric mismatch during training. By restoring HU fidelity, these DL-based CBCT to sCT techniques enable accurate daily dose recalculation, adaptive replanning, and auto contouring, transforming CBCT from a positioning aid into a quantitative backbone for modern image-guided radiotherapy.

### 6.3 Summary

Generative image synthesis is moving from proof of concept to a practical enabler in RT. More broadly, machine learning is reshaping the field, but clinical adoption must confront persistent risks of overfitting and domain shift. In radiation oncology, accuracy alone is not enough: even small rates of false negatives or false positives can have serious consequences. Addressing these risks requires rigorous validation, uncertainty reporting, and continuous quality assurance, with medical physicists playing a central role in understanding model limitations, monitoring performance, and integrating these rapidly evolving tools into safe, reliable workflows.

## 7 Cherenkov radiation imaging: emerging applications in modern RT

Cherenkov radiation emerges when charged particles traverse dielectric media at velocities exceeding the local speed of light. This phenomenon, characterized by its distinctive blue glow, occurs across the electromagnetic spectrum from ultraviolet to near-infrared wavelengths. In RT contexts, Cherenkov emission is generated whenever high-energy radiation interacts with tissue or water-equivalent phantoms, making it an intrinsic component of dose delivery processes (164–166).

The fundamental physics governing Cherenkov production follows well-established principles. The threshold condition requires β≥ 1/n, where β represents the particle velocity relative to light speed and n denotes the medium's refractive index. For electrons in liquid water, this threshold corresponds to approximately 260 MeV, with characteristic emission angles of ~41°. The Frank–Tamm formula describes Cherenkov intensity as proportional to 1/λ (2) in the wavelength domain, resulting in the characteristic-blue-weighted spectrum (167).

Under conditions of transient charged particle equilibrium, local Cherenkov intensity demonstrates strong proportionality to absorbed dose for both photon and electron beams (168–171). The presence of Cherenkov emission from radiotherapeutic proton beams has also been investigated (172). However, this relationship becomes complex due to factors including beam quality variations, spectral changes from beam hardening, and anisotropic secondary particle distributions. Furthermore, for *in vivo* applications, the optical transport of Cherenkov photons is dependent on patient-specific spatially heterogeneous tissue optical properties. These complications necessitate correction methodologies for accurate dosimetric applications.

### 7.1 Physics and detection considerations

The anisotropic nature of Cherenkov emission presents both challenges and opportunities for RT applications. In transparent media like water, Monte Carlo simulations and polarization imaging techniques can provide corrections to account for directional dependencies (169, 173). Alternatively, fluorophore doping can convert anisotropic Cherenkov light to more isotropic fluorescence, simplifying measurements while maintaining dose proportionality (174, 175).

In biological tissues, Cherenkov transport depends critically on optical properties, particularly absorption and scattering coefficients. The effective sampling depth is typically limited to several millimeters beneath tissue surfaces, with sensitivity decreasing exponentially with depth (164, 176–178). Unlike native Cherenkov spectra, tissue-emergent radiation exhibits red-shifted characteristics due to preferential absorption of shorter wavelengths (177).

Detection technologies have advanced significantly, enabling practical Cherenkov measurements in clinical environments. Modern systems employ intensified CMOS cameras to capture the relatively weak Cherenkov signals (on the level of μW cm-2 per Gy s^−1^ for external beam therapy), the sensitivity, and noise characteristics, which have been reported in the literature (179–183). Spectral filtering of ambient lighting and temporal gating synchronized to radiation pulses effectively suppresses ambient light interference, particularly valuable in low-duty cycle applications (184–186).

### 7.2 Dosimetric applications in phantom studies

Camera-based Cherenkov imaging has demonstrated significant utility for beam characterization and quality assurance in phantom studies. Two-dimensional projection imaging enables rapid profiling of electron and photon beams with excellent spatial resolution (165, 174). Tomographic reconstruction techniques allow three-dimensional dose distribution mapping, validated for intensity-modulated RT (IMRT) and volumetric modulated arc therapy (VMAT) quality assurance (187, 188).

The exceptional spatiotemporal resolution achievable with Cherenkov imaging addresses critical needs in modern RT. Advanced techniques, such as stereotactic radiosurgery, microbeam therapy, and stereotactic body RT, demand precise characterization of small fields with steep dose gradients. Cherenkov imaging provides sub-millimeter spatial resolution in both 2D projection and 3D tomographic modes (175, 189). These favorable properties have been leveraged in the applications of Cherenkov imaging to routing quality assurance, including for MR linacs (190–193).

Ultra-high dose rate (UHDR) RT, known as FLASH-RT, presents unique dosimetric challenges due to dose rates exceeding 40 Gy/s—several orders of magnitude above conventional delivery rates (~2–6 Gy/min). Traditional dosimeters often exhibit dose-rate dependencies that compromise accuracy under UHDR conditions. Cherenkov imaging, combined with fast electronics and feedback systems, has successfully addressed these challenges, enabling dosimetry, monitoring, and control applications in FLASH-RT (194–196).

### 7.3 Chemical and biological sensing applications

**Figure 7:**
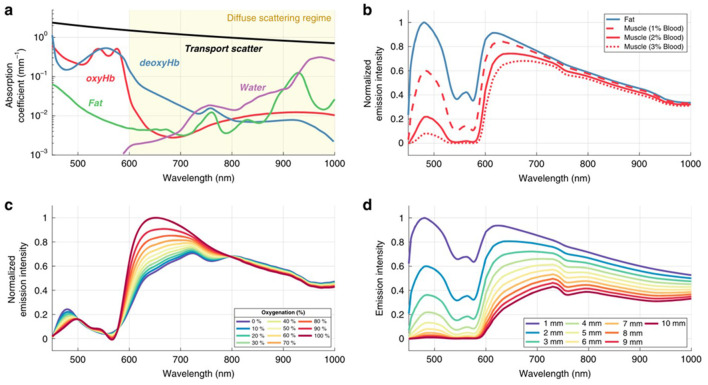
Spectral characteristics of tissue-emitted light are primarily determined by the absorption features of oxyhemoglobin, deoxyhemoglobin, water, and lipids, together with the general wavelength dependence of tissue scattering **(A)**. Simulated Cherenkov emission spectra originating from a 5 mm depth are shown for fatty tissue (composition: 90% fat, 9.5% water, 0.5% blood containing equal parts Hb and HbO_2_) and for radiodense tissue (mixtures of water and blood in varying ratios, again with equal Hb/HbO_2_ contribution) **(B)**. Changes in blood oxygenation within radiodense tissue containing 2% blood modify the Cherenkov spectrum owing to the distinct absorption profiles of oxy- and deoxyhemoglobin **(C)**. Finally, **(D)** illustrates how varying emission depth in radiodense tissue (2% blood) influences the resulting Cherenkov emission observed at the surface. Reprinted from Vasyltsiv et al. (200).

Cherenkov emission spectroscopy has emerged as a powerful tool for non-invasive chemical sensing, particularly for tissue oxygenation monitoring (197) (Figure 7). Conventional oxygen measurement techniques are often invasive and complex, limiting their clinical utility. Cherenkov-based approaches leverage spectral characteristics that correlate with tissue optical properties at varying oxygenation concentrations (198, 199). Multi-channel spectral Cherenkov imaging is an emerging technology that can provide additional contrast for subsurface features by leveraging the impact of tissue composition on the emitted Cherenkov spectrum (200). This approach was also used to generate the first color images of Cherenkov emission from patients (197).

The development of Cherenkov-excited luminescence techniques has expanded sensing capabilities beyond direct spectroscopic methods. By introducing oxygen-sensitive optical probes that can be stimulated by Cherenkov light, researchers have demonstrated real-time measurements of partial pressure of oxygen (pO_2_), both *in vitro* and *in vivo* (201, 202). Diffuse optical tomography with radiation beam-optimized excitation patterns enables three-dimensional oxygen distribution reconstruction to depths of several centimeters (203, 204).

Cherenkov-excited luminescence scanned imaging (CELSI) represents another jump forward in Cherenkov-based biological sensing. This technique utilizes two-dimensional radiation sheets to generate Cherenkov emission, which subsequently excites luminescence probes distributed throughout biological tissues. By incorporating prior knowledge of beam positioning, three-dimensional optical signal distributions can be reconstructed with high spatial resolution (205–207).

### 7.4 *In-vivo* clinical applications

Human Cherenkov imaging was first demonstrated in patients with breast cancer receiving external beam RT. Synchronization of frame capture with radiation pulses enabled real-time, background-subtracted imaging at rates exceeding 10 frames per second (166, 208). These proof-of-concept studies revealed field segments projected onto patient surfaces, with intensity correlations to subsequent erythema development.

Clinical applications have expanded to encompass multiple treatment sites and techniques, including total skin electron therapy, head and neck VMAT, and frame-based intracranial stereotactic radiosurgery (209–214). Primary applications focus on motion monitoring, coverage validation, and treatment verification, though quantitative dose correlation remains challenging due to patient-specific factors, such as tissue optical properties, beam geometry, and treatment modality.

Significant progress has been made in addressing quantitative limitations through patient-specific corrections, with a particular focus on breast RT. Spatial frequency domain imaging (SFDI) enables measurement of skin optical properties for Cherenkov intensity correction (215). Additionally, X-ray attenuation values extracted from planning CT scans show a strong correlation with optical absorption, providing an alternative correction approach that utilizes readily available imaging data (216, 217). The patient's skin tone has been incorporated into the correction paradigm by leveraging the intensity of the paired time-delayed images used for online Cherenkov-background subtraction (218). Cherenkov images have been used to monitor and analyze match line quality in half-beam blocked or multi-isocenter treatments (219–221), and there are ongoing attempts to utilize biological features in the images as fiducial markers to track setup accuracy, leveraging classical and deep learning-based image analysis techniques (222–224).

**Figure 8:**
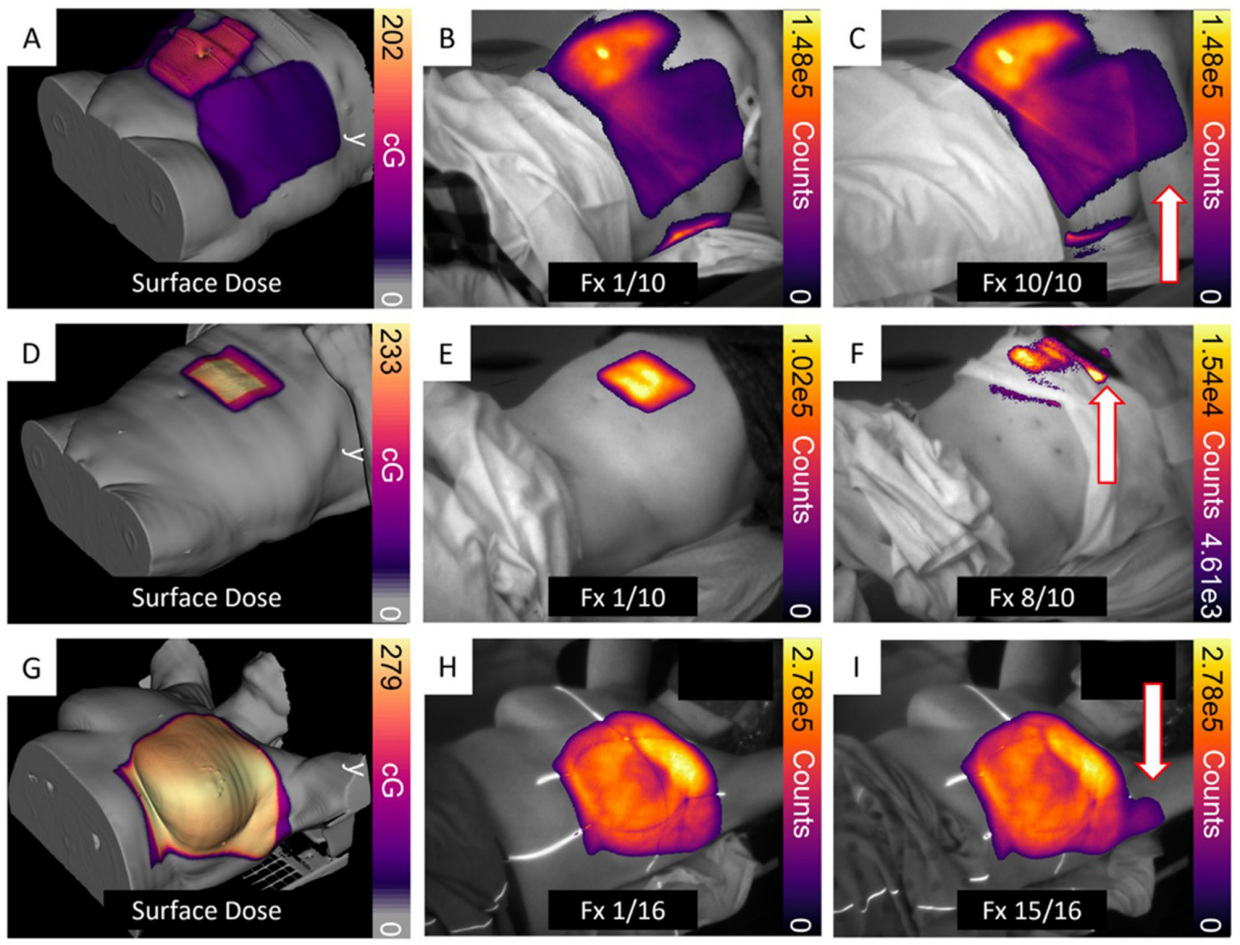
Examples of unintended dose delivery are illustrated for Cases 1, 2, and 3. (**A–C**, Case 1) depict incorrect arm positioning during 7 of 10 fractions, resulting in approximately 4.5 Gy exposure from an exit beam. (**D–F**, Case 2) show suboptimal hand placement that produced a small unintended dose in one of the ten fractions. (**G–I**, Case 3) demonstrate additional dose to the left axillary region caused by a slight displacement of the left arm in one of sixteen fractions. For all cases, surface-dose maps derived from the treatment plan were projected onto the corresponding patient CT surface, with red arrows marking the affected regions. Reprinted from Jarvis et al. (228).

Despite quantitative challenges, Cherenkov imaging offers unique advantages as a “free” signal present during any megavoltage RT without additional dose or time requirements. With the introduction of commercially available clinical Cherenkov imaging systems (BeamSite, DoseOptics, Lebanon NH, and DoseRT, Vision RT, London UK), there has been an effort to use the live Cherenkov video feed and the post-treatment cumulative image to monitor beam shape and improve or avoid non-ideal planning, delivery, or setup conditions (225, 226). Published studies on cohorts of 64 to over 600 patients have shown incident rates between 1.5% and 9% that were uniquely identified with Cherenkov imaging (227, 228) (Figure 8). Additionally, there are recent efforts to use Cherenkov images to guide the placement of *in vivo* dosimeters for surface dose measurements on the contralateral breast or verification of implanted electronic device dose limits (229, 230).

Future developments focus on automated anomaly detection through machine learning applications. The large-scale data availability from always-on Cherenkov imaging enables several promising applications, including deep image denoising, motion estimation, automated patient alignment verification, and real-time treatment anomaly detection (210, 211).

### 7.5 Emerging applications and future directions

FLASH-RT applications represent a rapidly expanding frontier for Cherenkov imaging. The instantaneous nature of Cherenkov emission makes it ideally suited for monitoring UHDR deliveries that typically occur within fractions of a second. Real-time Cherenkov imaging has been successfully demonstrated in large animal FLASH studies, providing quality assurance and delivery control capabilities (196, 231).

Advanced imaging techniques continue to evolve, including multi-spectral Cherenkov imaging for physiological parameter estimation. Time-gated, three-channel cameras have enabled color Cherenkov emission analysis, potentially providing information about oxygen saturation, blood volume, and tissue composition (197).

**Figure 9:**
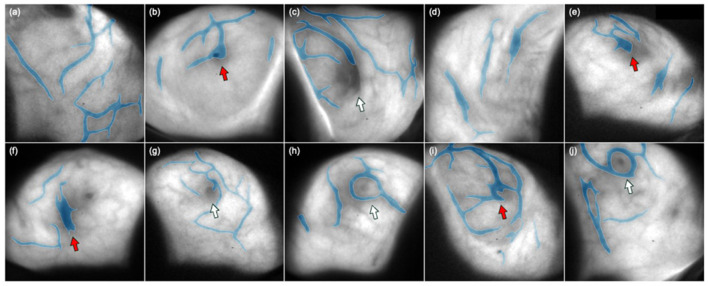
Visualization of segmented bio-morphological structures derived from Cherenkov imaging in ten representative breast cancer patients. **(A–J)** display the outputs of a fine-tuned SegResNet model, showing enhanced-edge segmentations of surface features overlaid transparently on the corresponding Cherenkov images. The segmented features primarily represent subcutaneous vasculature on the breast surface, with occasional inclusion of other anatomical details such as scars or nipples. Red arrows in **(B, E, F, I)** highlight segmentation errors involving scars and nipples, whereas white arrows in **(C, G, H, J)** indicate accurate segmentation results where vascular structures are correctly isolated. Reproduced with permission from Wang et al. (224).

Machine learning integration promises to enhance Cherenkov imaging capabilities significantly. Applications under development include automated treatment verification, real-time anomaly detection, patient-specific dose estimation, and physiological parameter extraction from spectral Cherenkov data (224, 232) (Figure 9).

### 7.6 Summary

Cherenkov radiation imaging has matured from a laboratory curiosity to a clinically viable technology with diverse applications in RT. While challenges remain in establishing quantitative dose correlations, particularly for *in vivo* applications, the technology offers unique advantages, such as real-time monitoring capabilities, excellent spatiotemporal resolution, and compatibility with emerging UHDR techniques. Continued technological development and clinical validation will likely expand Cherenkov imaging applications in quality assurance, biological monitoring, and treatment verification across conventional and advanced RT modalities.

## 8 Challenges in precision proton therapy

The popularity of proton therapy is derived from its capacity to spare healthy tissue while providing excellent dose conformity to the tumor due to proton physical properties, particularly the Bragg peak, where protons deposit their maximum energy at the end of their path at a precise depth. Thus, proton therapy is advantageous in pediatric oncology and tumors located near critical organs and structures. However, this unique characteristic comes as a double-edge sword: the precision of proton therapy is vulnerable to any uncertainties contained within the workflow of proton therapy. Even small inaccuracies during this process could lead to significant change in target coverage or unintended irradiation to the normal tissue.

**Figure 10:**
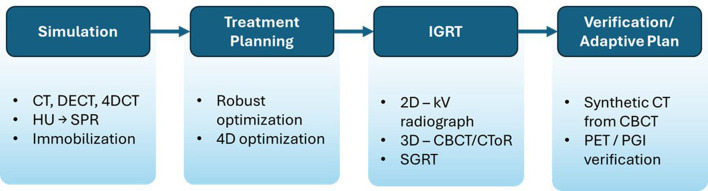
Imaging workflow in proton therapy.

Imaging plays an essential role in every step of proton therapy: from simulation, treatment planning, daily imaging-guided patient setup during treatment, and to potential adaptive planning (Figure 10). This section will first focus on imaging-related challenges in simulation and imaging guidance during daily treatment and then summarize emerging technologies and their future clinical implications.

### 8.1 Simulation imaging and planning accuracy

#### 8.1.1 Patient positioning and immobilization

Like photon-based treatments, proton therapy treatment begins with simulation, during which high-quality volumetric images, typically a CT scan, are acquired to define tumor target volumes and organs at risk (OARs) and used for dose calculation during treatment planning. Thus, reproducibility of patient setup during simulation is essential to ensure the patient position can be consistently and accurately recreated during treatments. Uncertainties introduced at this stage propagate throughout the entire workflow of the treatment.

Immobilization devices, such as thermoplastic masks for brain and head and neck tumors, VacLok^®^ for other sites, and indexed positioning systems, aid in minimizing variations in patient position. Special attention must be paid to all items that may vary in thickness and location. Changes of these items during the treatment course would significantly impact the dose distribution during daily treatment if placed within a beam path.

#### 8.1.2 CT HU to stopping power ratio (SPR) conversion

The cornerstone of proton therapy planning is the conversion of CT HUs to the stopping power ratio (SPR), which is used to calculate proton dose deposition. Uncertainties in this conversion are the major source of range uncertainty, which is estimated to be 2%−3% and more than 5% in the lung tissue (233, 234).

The standard clinical approach to address range uncertainty is the stoichiometric method, which involves acquiring single-energy CT (SECT) scans of various materials to establish a calibration curve. However, this curve is scanner- and protocol-dependent and assumes consistent image quality and minimal artifacts. Artifacts from metal implants or motion (e.g., respiration) introduce errors in SPR estimation, which ultimately propagate into dose calculation.

#### 8.1.3 Advanced CT imaging techniques

To improve SPR accuracy, the feasibility of using dual-energy CT (DECT) for proton treatment planning is being explored in research and clinical settings (235, 236). DECT acquires two images at different X-ray energies, enabling the calculation of effective atomic number and electron density. This provides a more accurate pathway to SPR estimation than the stoichiometric method. Studies have shown that DECT implementation reduces range uncertainty by more than 1% compared to conventional CT.

Four-dimensional CT (4DCT) is critical in thoracic and abdominal sites where the tumor moves due to respiration and peristalsis. 4DCT allows breathing phase-based sorting of images to capture motion and inform strategies, such as internal target volume (ITV) creation or beam gating. Nowadays, treatment planning system allows users to incorporate different breathing phases during optimization, which greatly reduces the impact of breathing motion on daily dose delivery (237, 238). Proper motion management strategy reduces inter- and intra-fractional motion while improving the robustness of the proton treatment plan.

### 8.2 Image guidance during treatment in proton therapy

#### 8.2.1 Imaging modalities

Table 2 compares different image modalities in proton therapy. Compared to photon therapy, which is inherently more forgiving for setup inaccuracies, proton therapy is highly sensitive to changes in water equivalent thickness (WET) along the proton beam path due to the finite range. Image-guided RT (IGRT) plays a vital role in verifying patient setup and minimizing uncertainties.

**Table 2:** Comparison of imaging modalities in proton therapy.

**Modality**	**Phase of use**	**Strengths**	**Limitations**
SECT (CT)	Simulation	Widely available; used for HU-SPR conversion	HU-to-SPR uncertainty; artifact sensitivity
DECT	Simulation	Better SPR accuracy; material decomposition	Limited clinical adoption; more complex workflow
4DCT	Simulation	Captures respiratory motion	Motion artifacts; longer simulation time
CBCT	IGRT/Verification	Volumetric guidance	Poor HU accuracy; not ideal for replanning; not widely available in all proton centers
CToR	IGRT/Verification	High-resolution, diagnostic-quality imaging	Set up shift required; space-consuming
pCT	Simulation	Direct SPR measurement	Still under development; resolution and workflow
MRI	Simulation/Planning	Excellent soft tissue contrast	No intrinsic SPR; image registration needed
PET	Verification	*In vivo* dose verification	Biological washout; logistical and temporal limits
Prompt-Gamma	Verification	Real-time range verification	Limited resolution; detector development ongoing
SGRT	IGRT	Non-ionizing; real-time monitoring	Limited to external surface

Common IGRT techniques include orthogonal kV radiographs and CBCT. Radiographs are quick and efficient for bony alignment but lack soft tissue visualization. CBCT offers volumetric information but suffers from lower image quality and longer acquisition time. Moreover, CBCT has poor HU accuracy, making it unreliable for proton range calculations (239). Nonetheless, it is superior in anatomy visualization, providing more reliable couch correction to align the anatomy on the treatment day with the simulation CT, reducing the setup uncertainties.

Surface-guided RT (SGRT) is an emerging modality used for patient positioning in superficial tumors or sites with minimal internal motion and for gating or breath-hold cases. It avoids ionizing radiation and provides real-time feedback. However, special attention needs to be paid when relying on SGRT to align the patient since it lacks internal anatomical correlation.

CT-on-Rails (CToR) has been adopted for IGRT modality in proton therapy to address the limitations of CBCT. The image quality of CBCT systems integrated into proton gantries is often compromised due to extended source-to-imager distance (SID) and limited mechanical clearance. In contrast, CToR provides diagnostic-quality volumetric imaging, offering superior soft tissue visualization and improved target delineation, which are requisite for accurate image registration and adaptive dose calculation. When implemented clinically, alignment between the CToR imaging isocenter and the treatment isocenter is critical. This alignment must be established during system installation and commissioning and maintained through routine quality assurance checks. Notably, during imaging, the treatment couch must be rotated or translated away from the treatment position to allow access to the CToR gantry—introducing the potential for setup variability if not properly managed.

#### 8.2.2 Adaptive proton therapy

Given the sensitivity of proton beams to anatomical changes, adaptive therapy is a growing area of interest. Adaptive strategies rely on periodic or daily imaging to assess the impact of changes in patient anatomy on dose delivery and adjust the treatment plan when deemed necessary. Recent research on synthetic CT generation from CBCT using deformable registration or AI techniques holds promise in overcoming its limitation of poor HU reliability (240). CToR images show superior HU accuracy, which would be readily used for adaptive proton treatment planning.

Adaptive therapy can improve target coverage and OAR sparing, particularly in long-course treatments. However, it requires additional clinical resources, including time for recontouring and plan evaluation, making routine implementation challenging.

### 8.3 Emerging technologies for imaging accuracy

#### 8.3.1 Proton radiography and proton CT

Table 3 summarizes the emerging image techniques for proton therapy. Proton radiography and proton CT (pCT) are imaging modalities that use protons themselves, rather than X-rays, to generate images of patient anatomy. Proton radiography provides 2D images and has been used in research settings for patient alignment and range verification (241). Proton CT, still under clinical development, offers 3D volumetric imaging and direct SPR mapping (242). Studies suggest that pCT can reduce range uncertainty to within 1%, though issues such as limited spatial resolution, longer acquisition times, and system integration remain barriers to clinical implementation.

**Table 3:** Emerging technologies and clinical readiness.

**Technology**	**Purpose**	**Advantages**	**Current limitations**	**Clinical maturity**
Dual-energy CT	Improve SPR estimation	Reduced range uncertainty	Needs new calibration and workflow	Moderate
Proton CT (pCT)	Direct SPR measurement	< 1% range uncertainty	Resolution, speed, system availability	Low (R&D)
Prompt gamma imaging	*In vivo* range verification	Real-time feedback	Detector complexity, resolution	Low–Moderate
Synthetic CT (from CBCT)	Adaptive planning	Enables CBCT-based replanning	Needs AI or deformable registration	Moderate
AI-based auto-segmentation	Planning/Adaptive	Efficiency, consistency	Validation and generalizability	Moderate
In-room PET	Post-treatment range verification	Biological dose imaging	Washout, timing, scanner access	Low

#### 8.3.2 Prompt gamma and PET imaging for in-vivo verification

Due to its finite range, it is impossible to measure the exit dose from proton delivery during treatment, compared to photon treatment. To verify proton beam delivery during or after treatment, prompt gamma imaging (PGI) and proton-induced PET are under active investigation.

PGI detects gamma photons emitted almost instantaneously as protons interact with nuclei in the patient. The spatial distribution of prompt gammas correlates with the proton range, offering a method for real-time verification. PGI systems are being tested clinically with encouraging results, though detector design and resolution constraints limit full clinical integration (243).

PET imaging leverages the positron-emitting isotopes generated by proton–nucleus interactions. Post-treatment PET can visualize areas where protons deposited energy. However, its clinical utility is limited by biological washout, low signal-to-noise ratios, and logistical challenges, such as the need for on-site PET scanners (243).

#### 8.3.3 Artificial intelligence in imaging accuracy

AI and ML are increasingly being applied to enhance imaging in proton therapy. Applications include:

- Synthetic CT generation from MRI or CBCT, enabling MR-based planning or CBCT-based adaptation with reliable HU/SPR mapping (244).
- Automated segmentation of targets and OARs, reducing variability and speeding up planning.
- Image registration improvements, particularly deformable registration across modalities.
- Artifact correction in CT and CBCT, especially for motion and metal-induced artifacts.

AI tools are being developed to predict anatomical changes and guide adaptive decision-making, potentially reducing the need for daily manual planning and review. As these tools mature, they could enhance the accuracy and efficiency of imaging workflows throughout the proton therapy process.

### 8.4 Summary

In summary, new technologies are improving the accuracy of proton therapy. Precision is limited by range uncertainty from HU-to-stopping-power conversion, setup/motion, and imaging artifacts. Dual-energy CT tightens SPR estimates, while 4DCT characterizes respiratory motion for robust planning. For image guidance, kV radiographs and CBCT improve daily alignment, SGRT provides non-ionizing motion monitoring, and CT-on-rails offers diagnostic-quality volumetric updates. Adaptive proton therapy leverages periodic or daily imaging to replan when anatomy changes. *In vivo* verification via prompt-gamma and PET supplies range feedback, and pCT can directly inform water-equivalent thickness modeling. AI further enables synthetic CT, automated segmentation, deformable registration, and artifact mitigation. Broad clinical impact will hinge on rigorous validation, standardized QA/reporting, interoperable data pipelines, and staffing/training to sustain adaptive, verification-rich practice.

## 9 Advanced imaging and dosimetry in theranostics

Radiopharmaceutical therapy (RPT) is a form of internal radiation treatment that combines tumor-targeting molecules with radioactive isotopes to deliver cytotoxic radiation directly to cancer cells. Unlike external beam radiotherapy (EBRT), which delivers radiation from outside the body, RPT administers radiation systemically, typically via the bloodstream, allowing it to target both primary tumors and metastatic sites. This targeted, systemic approach makes RPT particularly well-suited for treating widespread metastatic disease. When used in combination with other therapies, demonstrated promising efficacy and a favorable toxicity profile, often outperforming conventional RPT has systemic treatments in clinical trials (245, 246).

A defining feature of RPT is its compatibility with personalized medicine. As the distribution of radioactive agents can be visualized within the body using imaging—either after administration or through surrogate imaging—the treatment can be precisely tailored to each patient. This imaging capability enables real-time tracking of drug biodistribution and supports individualized dosing based on organ uptake and tumor burden. The term “theranostics” describes this integration of targeted therapy (thera-) with diagnostic imaging (-nostics) (247) and has led to a resurgence in the development and clinical use of RPT over the past two decades.

Theranostics involves the use of molecular imaging, typically with PET/CT or SPECT/CT, to guide patient selection and optimize treatment strategies. A common theranostic approach uses isotope pairs that are chemically identical but differ in their radio-physical properties, such as ^123^I/ ^124^I/ ^131^I and ^86^Y/ ^90^Y. In these cases, one isotope is used for imaging to assess biodistribution and receptor targeting, while the other is used for therapeutic radiation delivery. Additionally, theranostic approaches can utilize different elements that can be chelated to the same targeting molecules, such as ^68^Ga for imaging and ^177^Lu for therapy, when bound to identical targeting vectors like DOTA-peptides. An alternative strategy involves administering a trace amount of the therapeutic agent and imaging it directly using SPECT/CT to predict the drug's distribution during the treatment. These methods enable clinicians to better evaluate treatment feasibility and personalize RPT to achieve maximum efficacy with minimal toxicity.

### 9.1 Current RPTs administered in the clinic

Several RPTs have received FDA approval for treating a range of cancers, reflecting the growing role of targeted radionuclide therapy in oncology. Among the most widely used radionuclides in RPT are beta (β^−^)-emitters, such as iodine-131 (^131^I), yttrium-90 (^90^Y), and lutetium-177 (^177^Lu). These isotopes are favored for their tissue-penetrating radiation range and ability to induce DNA damage in tumor cells, ultimately leading to cell death.

One of the earliest and most established forms of RPT is radioactive iodine (RAI) therapy, which has been used clinically for nearly a century, particularly for the treatment of differentiated thyroid cancer and benign thyroid disorders such as Graves' disease and toxic multinodular goiter (248). RAI therapy exploits the sodium iodide symporter (NIS)—a transmembrane protein that facilitates active transport of iodide ions into thyroid follicular cells. This transporter is highly expressed in differentiated thyroid cancers, allowing for the selective accumulation of radioactive iodine within malignant tissues.

Beyond therapy, radioactive iodine isotopes are also used for diagnostic imaging. Sodium iodide labeled with ^123^I (Na^123^I) is commonly used for SPECT/CT imaging due to its favorable gamma photon energy and shorter half-life, which minimizes radiation dose to the patient. Na^124^I is a positron-emitting isotope of iodine, which is used for PET/CT imaging, providing higher spatial resolution and quantitative accuracy (249, 250).

Radium-223 dichloride (^223^RaCl_2_) was approved by the FDA in 2013 for the treatment of castration-resistant prostate cancer (CRPC) with symptomatic bone metastases. As an alkaline earth metal, ^223^RaCl_2_ mimics calcium and selectively localizes to areas of increased bone turnover, particularly at sites of metastatic lesions. Patients with metastatic prostate cancer often exhibit elevated bone remodeling activity driven by osteoblasts and osteoclasts, making them ideal candidates for therapies involving calcium mimetics, such as ^223^RaCl_2_ (251).

^223^Ra undergoes a six-stage decay process, emitting four alpha particles per decay. These alpha particles account for approximately 95% of the total decay energy, making ^223^Ra a highly potent source of localized radiation. The emitted alpha particles have high linear energy transfer (LET), which means they deposit a substantial amount of energy along short tracks. This results in efficient induction of DNA double-strand breaks, which are lethal to tumor cells. However, due to their short range (approximately 10–100 μm), the cytotoxic effects of alpha particles are confined to a radius of 2–10 cells, thereby minimizing damage to surrounding healthy tissue.

Meta-iodo-benzyl-guanidine (mIBG) is a norepinephrine analog that targets the adrenergic tissue. When labeled with iodine-131 (^131^I), mIBG has been used for decades to treat neuroblastoma and other pediatric tumors (252). Despite its long history of clinical use, standard ^131^I -mIBG does not have formal FDA approval and is therefore prescribed by physicians under investigational or compassionate use protocols (253). One should keep in mind that the prescribed activity for ^131^I-mIBG is based on patient body weight and is typically much higher than other RPT drugs since pediatric patients can better tolerate bone marrow suppression and can also receive stem cell support following treatment.

More recently, a new formulation known as high-specific-activity (HSA) ^131^I-MIBG has been developed. This version features a much higher proportion of the mIBG molecules labeled with ^131^I, significantly increasing its specific activity—the amount of radioactivity per unit mass of drug (254). In standard ^131^I–mIBG preparations, only about 1% of mIBG molecules are radiolabeled (~123.3 MBq/mg), whereas in HSA ^131^I–mIBG, nearly 100% of the molecules are labeled (~92,500 MBq/mg) (255). HSA ^131^I–mIBG received FDA approval in 2018 for the treatment of patients with locally advanced or metastatic pheochromocytoma or paraganglioma who require systemic anticancer therapy. However, the manufacturer of HSA ^131^I–mIBG discontinued production of the drug in 2023.

^177^Lu-DOTATATE was approved by the FDA in 2018 for the treatment of gastroenteropancreatic neuroendocrine tumors (GEP-NETs), which are neuroendocrine tumors originating in the pancreas or gastrointestinal tract. GEP-NETs are the most common subtype of well-differentiated neuroendocrine tumors, accounting for more than 70% of cases (256–259). ^177^Lu-DOTATATE targets somatostatin receptors, which are overexpressed in GEP-NETs. The radiopharmaceutical consists of a somatostatin receptor agonist (SSA), the chelator DOTA, and the therapeutic radionuclide ^177^Lu (259). To identify appropriate candidates for treatment, the companion diagnostic ^68^Ga-DOTATATE is used for PET imaging to confirm somatostatin receptor expression in tumors. The standard prescribed activity of ^177^Lu-DOTATATE is 7.4 GBq, which is administered every 8 weeks for a total of four doses.

The FDA approved ^177^Lu-PSMA-617 in 2022 for the treatment of castration-resistant prostate cancers (CRPCs) in patients whose tumors overexpress the transmembrane protein prostate-specific membrane antigen (PSMA). PSMA is an ideal therapeutic target due to its high expression on prostate cancer cells and its cell surface localization, which makes it readily accessible to targeted agents. PSMA-617 is a small-molecule PSMA inhibitor that binds specifically to this protein, allowing for targeted delivery of the radioactive isotope ^177^Lu to cancer cells (260).

The FDA has also approved multiple PSMA-targeted PET radiotracers for identifying patients eligible for ^177^Lu-PSMA-617 therapy by detecting PSMA expression in tumors. These companion diagnostics are critical in guiding treatment decisions and selecting appropriate candidates for radioligand therapy. Among these, ^68^Ga-gozetotide (also known as ^68^Ga-PSMA-11) was the first to receive FDA approval in 2020 for use in PSMA PET/CT imaging. It remains the most extensively studied and widely used radiotracer for PSMA-targeted imaging. In addition, the FDA approved two ^18^F-labeled tracers: [^18^F]DCFPyL in 2021 and [^18^F]rhPSMA-7 in 2023, further expanding the toolbox of PSMA-targeted imaging agents. These radiotracers offer advantages in image resolution and logistical flexibility due to the longer half-life of ^18^F compared to ^68^Ga.

Interestingly, the prescribed activity of ^177^Lu-PSMA-617 is 7.4 GBq per cycle, which matches the dose used for ^177^Lu-DOTATATE despite targeting entirely different tumors. The treatment is administered every 6 weeks, for up to six cycles, or until disease progression or unacceptable toxicity occurs.

It is increasingly evident that radiopharmaceutical therapy (RPT) is becoming a viable treatment option for a wide range of late-stage cancers. In some cases, RPT may also be used earlier in the course of disease, either as a standalone treatment or in combination with other therapeutic agents. Notably, with the exception of ^131^I–mIBG and HSA ^131^I–mIBG, the toxicity profiles observed in clinical trials of approved RPT agents are substantially lower than those associated with many conventional cancer therapies. This suggests that a significant proportion of patients may be clinically underdosed, highlighting an opportunity to optimize treatment delivery. All currently approved RPT agents emit radiation that can be imaged using SPECT/CT, enabling patient-specific dosimetry. This capability allows clinicians to personalize the administered activity for each patient based on their individual biodistribution and organ sensitivity, which is an approach that can be leveraged to maximize therapeutic efficacy while minimizing toxicity.

### 9.2 Advanced quantitative imaging in radiopharmaceutical therapy

Theranostics relies heavily on advanced imaging modalities to guide and monitor treatment with radiopharmaceuticals. The most commonly used imaging techniques in RPT are positron PET/CT and SPECT/CT. PET/CT, often using ^68^Ga- or ^18^F-labeled tracers, offers high sensitivity and spatial resolution for detecting molecular targets such as PSMA or somatostatin receptors, enabling precise patient selection and treatment planning. SPECT/CT, used with gamma-emitting isotopes, such as ^131^I or ^177^Lu, allows for real-time visualization of therapeutic agents and supports quantitative dosimetry to tailor treatment to individual patients. These hybrid imaging techniques not only confirm target expression before therapy but also assess biodistribution, monitor therapeutic response, and detect toxicity—making them essential tools in the practice of personalized medicine within RPT and nuclear oncology.

#### 9.2.1 PET/CT

PET/CT imaging is an advanced medical imaging technique that combines the functional insights of PET with the detailed anatomical information of CT. By integrating these two modalities into a single imaging session, PET/CT provides a comprehensive view of both physiological activity and structural abnormalities within the body. This dual capability has made PET/CT an essential tool in clinical practice, enhancing diagnostic accuracy, guiding treatment planning, and improving overall patient management. The technology continues to advance, with ongoing research aimed at improving image quality, optimizing protocols, and expanding its clinical applications.

PET/CT plays a central role in theranostics by enabling both the selection of appropriate patients for radiopharmaceutical therapy (RPT) and the personalization of treatment. One of its most critical uses is identifying whether a patient's tumor expresses the molecular target required for specific RPTs. For example, ^68^Ga-PSMA PET/CT is used to detect prostate-specific membrane antigen (PSMA) expression in patients with metastatic prostate cancer, guiding the use of ^177^Lu-PSMA-617 therapy. Similarly, ^68^Ga-DOTATATE PET/CT is used to confirm somatostatin receptor expression in patients with neuroendocrine tumors before initiating ^177^Lu-DOTATATE treatment.

Beyond patient selection, PET/CT is also valuable for treatment planning and dosimetry. By using diagnostic isotopes such as ^68^Ga or ^18^F, clinicians can estimate how therapeutic agents like ^177^Lu or ^90^Y will distribute throughout the body. This information allows for patient-specific dosimetry calculations, which help determine the optimal therapeutic dose while minimizing toxicity to healthy tissues. PET/CT is also routinely used to monitor treatment response by measuring changes in tracer uptake over time. A decrease in uptake on follow-up PET/CT scans can indicate a reduction in tumor activity or burden, supporting continued therapy or adjustment of the treatment plan.

Additionally, PET/CT enables early detection of disease progression or recurrence, often before structural changes are evident. For instance, ^68^Ga-PSMA PET/CT is highly sensitive for identifying biochemical recurrence in prostate cancer, even at low PSA levels, allowing for earlier intervention. Finally, PET/CT can help evaluate off-target uptake, such as accumulation in the kidneys, salivary glands, or bone marrow, which may signal potential toxicity risks. This information is essential for refining treatment protocols and protecting critical organs. Overall, PET/CT is a cornerstone of theranostic practice, offering a combination of molecular insight and anatomical precision to guide effective and personalized cancer care.

#### 9.2.2 SPECT/CT

SPECT/CT imaging is a hybrid imaging technique that combines the molecular imaging capabilities of single photon emission computed tomography (SPECT) with the anatomical precision of CT. This integration allows for the simultaneous assessment of functional processes and structural features within the body, enhancing the localization and interpretation of radiopharmaceutical uptake. SPECT/CT has become a valuable tool in clinical practice, particularly in oncology, cardiology, and endocrinology, where it supports accurate diagnosis, guides therapeutic decisions, and aids in treatment response monitoring. Its utility in radiopharmaceutical therapy (RPT) is especially notable, as it enables real-time visualization of therapeutic agent distribution and facilitates quantitative dosimetry. Continuous advancements in detector technology, image reconstruction algorithms, and radiotracer development are further expanding the clinical applications and diagnostic performance of SPECT/CT.

SPECT/CT plays a critical role in theranostics by enabling both the visualization and quantification of radiopharmaceutical distribution, particularly for therapies involving gamma-emitting isotopes. One of its key uses is in patient-specific dosimetry for radiopharmaceutical therapies such as ^131^I, ^177^Lu-DOTATATE, and ^223^RaCl_2_ (298). By providing three-dimensional functional imaging overlaid with anatomical detail, SPECT/CT allows clinicians to assess how the therapeutic agent distributes across tumors and normal organs, enabling precise calculation of absorbed radiation doses. This supports personalized treatment planning aimed at maximizing efficacy while minimizing toxicity. SPECT/CT is also used to monitor treatment response by evaluating changes in radiotracer uptake over time, which can indicate tumor regression or progression. In some cases, a trace amount of the therapeutic agent is administered and imaged with SPECT/CT prior to full-dose therapy to predict biodistribution and assess treatment feasibility. Additionally, SPECT/CT can identify off-target uptake, helping to detect and mitigate potential risks to critical organs such as the kidneys, salivary glands, or bone marrow. Its accessibility, compatibility with a wide range of therapeutic isotopes, and ability to support real-time imaging of therapy delivery make SPECT/CT an important tool in the theranostic workflow.

### 9.3 Challenges with quantitative imaging

Accurate quantitative PET/CT or SPECT/CT imaging is essential in theranostics because it directly informs critical aspects of personalized treatment planning and clinical decision-making. In theranostics, imaging is not only used for diagnosis and staging but also to measure the *in vivo* distribution of radiopharmaceuticals, enabling patient-specific dosimetry. Precise quantification allows clinicians to calculate the absorbed radiation doses to tumors and normal organs, which is key to balancing efficacy with safety. Inaccurate quantification could lead to underdosing, which reduces therapeutic effectiveness, or overdosing, which increases the risk of toxicity to healthy tissues.

Moreover, quantitative imaging is vital for monitoring treatment response. Changes in standardized uptake values (SUVs) or other quantitative metrics over time provide objective evidence of how well a tumor is responding to therapy. This helps guide decisions about whether to continue, adjust, or stop treatment. It is also crucial for assessing biodistribution in advance of therapy, especially when using a diagnostic surrogate or microdose of the therapeutic agent to predict how the full treatment will behave. Without accurate quantitative imaging, these predictive models become unreliable.

Finally, quantitative PET/CT and SPECT/CT play an important role in clinical research and regulatory approval, where reproducible, measurable outcomes are needed to validate new theranostic agents and protocols. In short, accuracy in quantitative imaging underpins the safety, effectiveness, and precision that define modern theranostic approaches.

Quantifying the distribution of radiopharmaceutical activity within the body is a foundational step in theranostics, as it directly informs absorbed dose calculations and guides patient-specific treatment planning and treatment response assessment. However, achieving accurate quantification is inherently complex and subject to multiple sources of uncertainty. These include limitations in imaging system resolution and sensitivity, patient movement, image noise, and challenges in correcting photon attenuation and scatter. Additionally, variability in segmentation, registration, and calibration processes further complicates the measurement of activity, particularly in small regions of interest, with a heterogeneous uptake (e.g., lesions). Understanding and addressing these sources of uncertainty is critical for improving the accuracy, reproducibility, and clinical utility of quantitative imaging in theranostics. The following sections explore the major technical and procedural factors that contribute to uncertainty in activity quantification.

#### 9.3.1 Quantification of activity

Uncertainty in quantifying activity distribution refers to the challenges and potential sources of error in measuring how a radiopharmaceutical is distributed within the body, especially within specific organs, tissues, or lesions. Accurate quantification is essential in theranostics because it directly impacts dosimetry calculations and, ultimately, the determination of the absorbed radiation dose. Several factors contribute to this uncertainty, including the limited spatial resolution and sensitivity of the imaging system, partial volume effects (which can lead to underestimation of activity in small structures), and inaccuracies in attenuation and scatter correction. Calibration errors in the imaging system can also affect the reliability of activity measurements. Reducing these uncertainties is critical to ensure precise, patient-specific treatment planning in theranostics.

*Spatial resolution:* Imaging systems such as SPECT and PET have limited spatial resolution, which means they cannot accurately differentiate fine details in small structures. As a result, activity within small organs or lesions may be underestimated due to the partial volume effect (PVE)—a phenomenon where the true activity appears diluted across neighboring voxels, making small structures appear less intense than they actually are. This loss of detail leads to PVE-related inaccuracies in the reconstructed images, which is a well-known limitation of nuclear medicine cameras (261–264).

*Sensitivity:* The sensitivity of imaging systems varies and refers to the ability of PET or SPECT scanners to detect photons emitted by the radiopharmaceutical. However, no system detects all emitted photons perfectly. Some photons are scattered, absorbed, or missed entirely, which can result in underestimation of total activity and contribute to quantification errors (255, 265, 266).

*Noise in Imaging*: The signal-to-noise ratio (SNR) plays a critical role in the accuracy of quantitative imaging. Noise can arise from several factors, including limited scan duration, low radiopharmaceutical dose, and patient movement. High noise levels, particularly in areas of low radiotracer uptake (such as surrounding healthy tissue), make it difficult to accurately measure activity. Although increasing scan time and applying noise reduction techniques can improve accuracy, these solutions may reduce patient comfort and limit scanner throughput (255).

*Patient motion:* Motion during image acquisition, such as respiratory or involuntary movement, can blur the observed activity distribution and introduce quantification errors. This is especially problematic when imaging small structures. For example, one simulation study using ^90^Y bremsstrahlung SPECT showed that respiratory motion reduced the recovery coefficient of a tumor from 90% to 66% (267, 268).

*Calibration:* Accurate activity quantification depends on proper calibration of the imaging system, which involves translating detected photon counts into units of radioactivity. This is typically done using radioactive phantoms with known activity distributions. The system is then adjusted to match these known values, establishing a reference for interpreting patient scans. Calibration errors—caused by system performance variability, improper calibration procedures, or aging hardware—can significantly impact quantitative accuracy (255, 269).

#### 9.3.2 Attenuation and scatter correction

During nuclear imaging, photons emitted from radiopharmaceuticals can be absorbed or scattered by tissues as they pass through the body. This process, known as attenuation, is particularly pronounced in denser structures like bone or organs and can lead to underestimation of activity in deeper tissues if not properly corrected. While attenuation correction algorithms are routinely applied, they introduce uncertainty—especially when the patient's anatomy deviates from standard models.

Photon scatter further complicates quantification by reducing image contrast. Scattered photons contribute a diffuse background signal, which can overestimate activity in low-uptake regions and underestimate activity in high-uptake areas like tumors (269). Various scatter correction methods are used to address this, but all introduce potential sources of error depending on imaging conditions, radiopharmaceutical properties, and patient-specific factors (255, 269).

In SPECT imaging, energy window-based methods such as double energy window (DEW) and triple energy window (TEW) are commonly used. These approaches estimate scatter from adjacent energy windows and subtract it from the primary signal. However, their accuracy depends on proper window placement and assumptions about scatter distribution. Misestimation can result in over- or under-correction, affecting final quantification (270–274). For instance, phantom studies have shown that TEW improves contrast-to-noise ratio over DEW in ^131^I and ^177^Lu SPECT, but may produce lower recovery coefficients, suggesting underestimation of true activity (275, 276).

More advanced approaches, such as Monte Carlo (MC)-based scatter correction, simulate individual photon interactions within the patient using detailed physical models (277, 278). These methods account for tissue composition, density, and photon transport, providing more accurate scatter estimation. Comparative studies have shown that MC methods outperform TEW for radionuclides like ^99m^Tc, ^111^In, and ^177^Lu, with TEW overestimating activity by up to 11% in ^177^Lu imaging due to its inability to capture patient-specific activity distribution (277, 279, 280).

#### 9.3.3 Partial volume effects

The partial volume effect (PVE) occurs when the spatial resolution of an imaging system is insufficient to accurately capture activity within small structures. As a result, activity appears blurred between adjacent regions, leading to underestimation in small, high-uptake areas (e.g., tumors or lymph nodes) and potential overestimation in surrounding low-uptake tissues (261, 262). Structures such as the thyroid, bone marrow, and small tumors are especially vulnerable because their dimensions often fall below the system's resolution, causing activity “spillover” into adjacent areas.

In addition to size, heterogeneous radiopharmaceutical uptake within tumors or organs can be distorted by PVE, obscuring the true distribution of activity. The magnitude of this effect is often characterized using recovery coefficients (RCs), which are derived from phantom studies that measure how much of the true activity is recovered in structures of various sizes and positions. RCs can vary widely—from below 0.1 to above 0.9—depending on factors such as isotope, object size, scanner type, and imaging settings (281). Placement within the field of view also affects RCs; one study using ^177^Lu demonstrated significantly different RCs for the same sphere size depending on its location in the phantom (281).

To better assess PVE in anatomically relevant settings, anthropomorphic phantoms have been developed for organs such as the kidney (262, 282), liver (283), and the head and neck region (40), allowing more realistic estimation of recovery in complex geometries.

#### 9.3.4 Segmentation

Accurate volume delineation is a critical yet time-consuming step in radiopharmaceutical therapy (RPT) dosimetry and is increasingly complex due to the need to register and interpret multi-timepoint and multimodality imaging (263, 284–286). Inter-observer variability (IOV) in segmentation is widely recognized as the largest source of uncertainty in the dosimetry process (287), potentially impacting both treatment efficacy and toxicity, as well as consistency across clinical centers.

Empirical studies have assessed the impact of segmentation variability by applying controlled changes (e.g., expansions or contractions) to segmented regions of interest (ROIs) and observing the resulting variability in mean absorbed dose (288, 289). These studies show that for organs and large tumors, contour variability is the dominant source of uncertainty, while for small tumors, sensitivity to the recovery coefficient becomes more significant.

Another approach involves directly comparing contours generated by multiple observers. For example, a recent Society of Nuclear Medicine and Molecular Imaging (SNMMI) “Dosimetry Challenge” analyzed dose estimates from 178 participants using common ^177^Lu-DOTATATE patient data (290–292). The study found segmentation to be a major contributor to dose variability, with normalized activity variability in healthy organs at 7% and lesion variability ranging from 6.7% to 24% (293). These findings support the development of standardized segmentation guidelines to reduce variability and improve the accuracy and reproducibility of RPT dosimetry.

#### 9.3.5 Registration

Accurate image registration is essential for reliable dosimetry in radiopharmaceutical therapy (RPT), yet it remains an underexplored area in the literature. Studies have shown that even small misregistrations—such as translations under 9 mm or rotations under 5°–can cause absorbed dose errors of up to 90% in tumor regions, especially when lesions are located away from the center of the SPECT field of view (294–297). These findings underscore the sensitivity of dose calculations to registration accuracy, particularly in tumor volumes.

While early studies focused on SPECT-only datasets, more recent research has evaluated registration techniques in multi-timepoint SPECT/CT. Comparisons between rigid and non-rigid (deformable) registration methods consistently show that non-rigid approaches provide greater alignment accuracy, especially in complex datasets. CT-based registration—where the CT images guide alignment and the corresponding SPECT data is adjusted—has shown better consistency in activity quantification than SPECT-based methods (296).

Simulated phantom studies further highlight the benefits of non-rigid registration, showing substantial reductions in alignment errors. For instance, spleen and liver misalignments dropped from 15.5% to 2.1% and from 7.3% to 0.2%, respectively, when using deformable registration instead of rigid methods (297). Patient studies echo these findings: in ^177^Lu-DOTATATE therapy, deformable registration resulted in higher absorbed dose estimates compared to rigid registration, with differences in kidney dose ranging from −19% to 4% and in tumor dose from −67.2% to 100.7% (295).

Proper patient positioning is also critical during multi-timepoint imaging. Movement between scans introduces alignment errors that can persist even after registration, leading to further uncertainties in dosimetry. These findings emphasize the need for careful registration method selection and consistent patient positioning to improve the accuracy of dose estimates in RPT.

### 9.4 Summary

Radiopharmaceutical therapy (RPT) is an increasingly important modality in oncology, offering targeted, systemic radiation delivery using tumor-seeking molecules labeled with radioactive isotopes. Unlike external beam radiotherapy, RPT can treat both primary and metastatic disease sites with relatively low toxicity profiles, making it a promising option for patients with late-stage or refractory cancers. A hallmark of RPT is its integration into theranostics—combining diagnostic imaging with therapy to enable personalized treatment planning based on patient-specific biodistribution and molecular target expression.

Multiple RPT agents have gained FDA approval, including ^131^I for thyroid cancer, ^223^RaCl_2_ for prostate cancer with bone metastases, ^177^Lu-DOTATATE for neuroendocrine tumors, and ^177^Lu-PSMA-617 for prostate cancer. These therapies leverage diagnostic counterparts, such as ^68^Ga- or ^18^F-labeled PET tracers, to guide patient selection and assess target expression. Quantitative imaging using PET/CT and SPECT/CT plays a pivotal role in RPT by enabling individualized dosimetry, monitoring therapeutic response, and identifying potential off-target toxicity.

However, accurate quantification of radiopharmaceutical distribution remains technically complex. Sources of uncertainty include limited spatial resolution, sensitivity loss, partial volume effects, attenuation and scatter artifacts, segmentation variability, and image registration inaccuracies. Advances in imaging technologies, standardized protocols, and sophisticated correction algorithms are essential to improve reproducibility and optimize treatment delivery. As RPT continues to evolve, the ability to reduce these uncertainties will be critical to fully realizing the potential of precision medicine in nuclear oncology.

## 10 Conclusion

In conclusion, emerging technologies are rapidly reshaping the landscape of radiation oncology. Across the RT workflow, advanced imaging is enabling finer target definition, smarter motion management, and increasingly adaptive, biology-informed dose delivery. MR-guided RT brings daily soft-tissue visualization and online adaptation; PET-guided strategies and integrated PET-linac concepts extend guidance to the molecular scale; stereoscopic X-ray with thermal surface guidance supports sub-millimeter CNS positioning; and CBCT-based online adaptation (e.g., HyperSight-enabled workflows) turns daily anatomy into actionable plans. In parallel, generative AI for image synthesis is shortening acquisition chains and improving quantitation, while Cherenkov imaging offers real-time treatment verification and new avenues for QA and FLASH monitoring. In proton therapy, better HU to SPR mapping through DECT, robust motion imaging (4DCT, CToR), and *in vivo* range verification (prompt-gamma, PET) are converging on tighter range uncertainty. Beyond external beam, theranostics couples diagnostic specificity with patient-specific dosimetry to personalize radiopharmaceutical therapy.

Realizing these gains at scale will require rigorous multicenter validation, standardized QA and reporting, integration of multi-omics with functional/quantitative imaging, and trustworthy automation with continuous performance monitoring. Equally important are interoperable data pipelines, workforce training (especially for medical physicists), and attention to access and equity so that precision benefits reach diverse patient populations. Together, these advances point toward safer, more adaptive, and genuinely personalized RT.
