# Correction: Innovative approaches in precision radiation oncology: advanced imaging technologies and challenges which shape the future of radiation therapy

**DOI:** 10.3389/fmed.2025.1738811

**Published:** 2025-11-25

**Authors:** Yue Yan, Daniel A. Alexander, Bryan P. Bednarz, Lawrence F. Bronk, Huixiao Chen, David J. Gladstone, Bin Han, Christopher M. Iannuzzi, Yuting Li, Ngoc Nguyen, Natasha Mulenga, Natalie N. Viscariello, Yuenan Wang, Joseph Weygand, Yana Zlateva, Fada Guan

**Affiliations:** 1 Department of Radiation Oncology & Applied Sciences, Dartmouth-Hitchcock Medical Center, Lebanon, NH, United States; 2 Geisel School of Medicine, Dartmouth College, Hanover, NH, United States; 3 Thayer School of Engineering, Dartmouth College, Hanover, NH, United States; 4 Department of Medical Physics, University of Wisconsin Madison, Madison, WI, United States; 5 Department of Radiation Physics, University of Texas MD Anderson Cancer Center, Houston, TX, United States; 6 Department of Therapeutic Radiology, Yale University School of Medicine, New Haven, CT, United States; 7 Department of Radiation Oncology, Stanford University, Stanford, CA, United States; 8 Hartford Health Care Cancer Institute, St. Vincent's Medical Center, Bridgeport, CT, United States; 9 Department of Radiation Oncology, Heersink School of Medicine, University of Alabama at Birmingham, Birmingham, AL, United States

**Keywords:** magnetic resonance imaging guided radiotherapy, positron emission tomography, stereoscopic imaging and surface guidance, cone beam computed tomography, generative image synthesis, Cherenkov radiation imaging, imaging innovations in proton therapy, advanced quantitative imaging

The reference in the caption of Figure 6 was erroneously cited as Maas et al. (104). It should be Ronneberger et al. (59).

The caption of Figure 6 has been updated to “Architecture of the U-Net. Reproduced with permission from Ronneberger et al. (59).”

The reference in the caption of Figure 9 was erroneously cited as Alexander et al. (227).

It should be Wang et al. (224).

The sentence “Reprinted from Alexander et al. (227).” has been corrected to “Reproduced with permission from Wang et al. (224).” in the caption of Figure 9.

Reference 224 was erroneously written as “Wang S, Chen Y, Jarvis LA, Tang Y, Gladstone DJ, Samkoe KS, et al. Robust Real-time segmentation of bio-morphological features in Human Cherenkov imaging during radiotherapy via deep learning. *arXiv [Preprint]. arXiv:2409.05666v1* (2014). doi: 10.1002/mp.18002”. It should be “Wang S, Chen Y, Jarvis LA, Tang Y, Gladstone DJ, Samkoe KS, et al. Robust real-time segmentation of bio-morphological features in human Cherenkov imaging during radiotherapy via deep learning. *Med Phys*. (2025) 52:e18002. doi: 10.1002/mp.18002.”

Reference 1 was incorrectly cited in section **6 Image synthesis in RT**, *6.1 Deep learning networks in medical images*, Paragraph 2. Reference 59 should have been cited.

The sentence previously read: “One of the most well-known CNN models is the U-shaped net (U-Net) proposed by Ronneberger et al. (1) (Figure 6).”

The sentence now reads: “One of the most well-known CNN models is the U-shaped net (U-Net) proposed by Ronneberger et al. (59) (Figure 6).”

The original version of this article has been updated.
